# Life expectancy among older adults with or without frailty in China: multistate modelling of a national longitudinal cohort study

**DOI:** 10.1186/s12916-023-02825-7

**Published:** 2023-03-16

**Authors:** Junling Gao, Yujie Wang, Jixiang Xu, Junjia Jiang, Shitong Yang, Qianyi Xiao

**Affiliations:** 1 School of Public Health, Fudan University, Shanghai, 200032 China; 2 Collaborative Innovation Cooperative Unit of National Clinical Research Center for Geriatric Diseases, Shanghai, 200032 China; 3 Core Unit of Shanghai Clinical Research Center for Aging and Medicine, Shanghai, 200040 China

**Keywords:** Life expectancy, Frailty, Older adult, Healthy aging, China

## Abstract

**Background:**

Little is known about life expectancy (LE) with or without frailty. We aimed to estimate the total LE and duration of the state of frailty in China.

**Methods:**

This study included older adults aged 65 years and older from the Chinese Longitudinal Healthy Longevity Study (CLHLS). Frailty status was classified into robust, pre-frailty and frailty based on a cumulative deficit model. Total and specific frailty state LEs at 65 years of age were estimated and stratified by demographic characteristics, behaviours, and psychosocial factors using continuous-time multistate modelling.

**Results:**

The total LE of older adults aged 65 years in China was 14.74 years on average (95% CI: 14.52–14.94), of which 4.18 years (95% CI: 4.05–4.30) were robust, 7.46 years (95% CI: 7.31–7.61) pre-frail and 3.10 years (95% CI: 3.01–3.20) frail. Older adults with higher robust LE included men (4.71 years, 95% CI: 4.56–4.88), married older adults (4.41 years, 95% CI: 4.27–4.56), those engaging in physical activity (4.41 years, 95% CI: 4.23–4.59), those consuming fruits daily (4.48 years, 95% CI: 4.22–4.77) and those with high social participation (4.39 years, 95% CI: 4.26–4.53). Increased educational attainment were gradually associated with increased robust LE.

**Conclusions:**

Frailty may lead to a reduced total LE and robust LE of older adults in China. In addition to finding inequalities in total and robust LEs by socioeconomic status, our findings also highlight that healthy behaviours and social participation may ease frailty-related reductions in total and robust LE. Our findings imply that national life-course strategies aimed at frailty screening and psychosocial and behavioural interventions could be important for health aging in China.

**Supplementary Information:**

The online version contains supplementary material available at 10.1186/s12916-023-02825-7.

## Background

Global life expectancy (LE) at 60 years of age has increased from 18.8 years in 2000 to 21.1 years in 2019. However, healthy life expectancy (HALE) at age 60 years has only risen from 14.1 to 15.8 years in the same period [1], leading to a slight increase in the number of years living with disability.

Frailty is a state of increased vulnerability to the poor resolution of homeostasis following stress [2], an emerging global health burden accompanying rapid population aging [3]. Evidence has demonstrated that frailty is associated with adverse outcomes, including hospitalization [4], falls [5], disabilities [6] and mortality [7]. Furthermore, frailty is a dynamically reversible entity; thus, it is possible to prevent or delay the onset of frailty and improve LE and HALE. There are two models for measuring frailty [2], the *phenotype model* and the *cumulative deficit model,* both of which classify frailty as robust, pre-frailty and frailty [7, 8]. To date, a few studies have estimated total LE with and without frailty using cross-sectional data by the Sullivan method based on the *phenotype model* [9–12]*.* However, the *phenotype model* does not include cognitive impairment, a highly prevalent condition associated with functional decline and disability [2]. The frailty index based on the *cumulative deficit model* is defined as the proportion of accumulated deficits (symptoms, signs, functional impairments, and laboratory abnormalities) [13] and is the most useful in routine care and community settings [14]. Studies have shown that the frailty index is a surrogate measure of biological age [7]; however, no study has estimated total LE and HALE based on the frailty index.

Of note, the Sullivan method is particularly practical, as it is usable for cross-sectional data, but it cannot capture the natural course of a disease [15]. A multistate model describes how individuals transition between different stages of a condition [16]. Given the dynamic nature of frailty, a multistate model can incorporate the fact that individuals move between frailty states over time, i.e., robust, pre-frailty and frailty, and estimate state-specific and total LE [17]. A recent study using a multistate model revealed that total and frailty-free LEs were 15.4 years and 14.1 years, respectively, among Brazilian adults aged 65 years [12].

As one of the countries with the fastest and largest aging population, China may face substantial challenges due to frailty, with a reported prevalence of 7.0%-15% [18] among various studies in older Chinese adults. In this study, we aimed to (i) examine transitions in frailty status and related factors and (ii) estimate total and specific frailty state LEs and their distributions among different groups in a 20-year prospective cohort study with a large, nationally representative sample of older adults in China.

## Methods

### Study participants

This study was based on the Chinese Longitudinal Healthy Longevity Study (CLHLS), which is an ongoing dynamic cohort study conducted among community-dwelling adults aged ≥ 65 years randomly selected from 22 provinces in China constituting approximately 82% of the total population in China in 2010. A more detailed description was published elsewhere [19]. Eight waves of surveys were conducted from 1998 to 2018. The biomedical ethics committee of Peking University approved the study (IRB00001052-13,074), and all participants or their respondents provided written informed consent.

A total of 43,205 participants were enrolled separately in 1998, 2000, 2002, 2005, 2008, 2011 and 2014 and were followed up with until 2018. Because the multistate model requires participants to respond to at least two waves of surveys to examine transitions in frailty status, 36,348 participants were included in the final analysis after excluding those aged < 65 years, those who responded to only one wave survey, or those with missing data on age or follow-up time (Additional file 1). The response rate was 84.13%.

### Measures

#### The frailty index (FI) and death

Following the standard procedure [20], we used 38 self-reported health deficits that were also used in a previous study [21], including psychological characteristics, activities of daily living (ADL), instrumental activities of daily living (IADL), hearing or vision impairment, cognitive functioning and chronic diseases, to construct the FI (Additional file 2). Each item was dichotomous or ordinal and measured on a scale of 0 to 1 to represent the severity of health deficits. We excluded deficits with missing information from both the denominator and the numerator. If data for over 30% of the deficits were missing, the FI was treated as missing. The FI was calculated using unweighted counts of the number of deficits divided by the total possible number of deficits. The frailty index score ranged from 0.00 to 1.00, with a higher value indicating a worse, frailer status. With reference to a previous study among older Chinese adults [22], we further categorized the frailty index into three levels of frailty: robust (frailty index score ≤ 0.10), pre-frailty (frailty index score > 0.10 to ≤ 0.21), and frailty (frailty index score > 0.21).

Information on death was collected from the next of kin (children or spouse) or the primary caregiver. In addition, the date and cause of death were confirmed using the death certificate, and another confirmation was typically acquired from the local neighbourhood committee if the death certificate was not available [23]; thus, the data quality was good. The duration of follow-up was calculated as the time interval between the first interview date and the date of death. Survivors at the wave after which they were last surveyed were considered censored at the time of the survey.

**Fig. 1:**
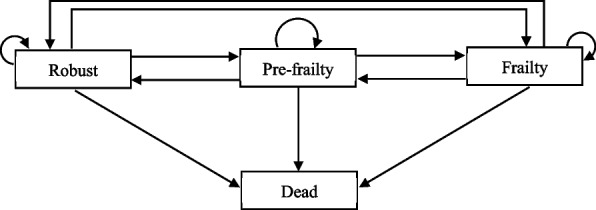
Multistate model of transitions of frail states

At each wave, respondents were classified as deceased or into one of three frailty states (robust, pre-frailty and frailty) (Fig. 1).

#### Subpopulation identifiers

To examine potential factors related to transitions in frailty status and total LE and frailty-specific LE (robust LE, pre-frailty LE and frailty LE), we classified the whole population into subpopulations by sex, educational attainment, marital status, occupational types, health behaviours (smoking, drinking, physical activity, fruit intake and vegetable intake), and region based on the literature [24, 25]. Sex (male or female), educational attainment (coded as 0 years, 1–5 years, 6–11 years and ≥ 12 years), marital status (married or unmarried), and health behaviours were identified by self-reported data in the earliest response. Smoking, drinking, and physical activity were measured by asking the participants to respond to a single yes/no question: “Do you smoke/drink/exercise or not at present?”. Those who responded “yes” were considered to be performing the corresponding behaviours. Daily fruit and vegetable intake were assessed by asking the following question: “How often do you eat fresh fruit/vegetables,” which was answered using the following scale: “almost every day”, “except winter”, “occasionally” and “rarely” or “never”. Those who answered “almost every day” were coded as “regularly”, while those who answered “except winter”, “occasionally” and “rarely” or “never” were coded as irregularly.

The region was identified according to government office region (East China, Central China, West China, and Northeast China) using *zip* codes in the database.

In addition, we examined occupational types and social participation (Additional file 3), two psychosocial factors that affect total and frailty-specific LEs.

### Statistical methods

First, to examine the potential factors related to transitions in frailty status, as defined in Fig. 1, a general multistate Markov model with covariates (subpopulation identifiers) [26] was conducted by the *msm* package [27] in R language [28]. Hazard ratios (HRs) and 95% confidence intervals (95% CIs) for each potential factor were reported.

Second, continuous-time multistate models were used to estimate total and frailty-specific LE [29] in two steps (Additional file 4). In the first step, a multinomial regression was fitted to estimate the yearly probability of a transition from any starting state (e.g., robust or frailty) to any outcome state (e.g., pre-frailty, frailty, or death) across any two waves of data. In the second step, the yearly transition probabilities obtained from the first step were used as input for a multistate life table (MSLT) method to calculate total and frailty-specific LE (robust LE, pre-frailty LE and frailty LE) by the *elect* (*Estimating Life Expectancies in Continuous Time)* package of R [17]. In this study, we assumed that the maximal human age was 120 years old [30] when we ran *elect* with the default “*step*” method for the numerical approximation. For each estimate, a standard error was calculated by a bootstrapping method that executed 500 repeated estimates through random draws. Separate analyses were performed for each subpopulation identifier.

### Sensitivity analysis

Five methods of sensitivity analysis were used to assess the robustness of the findings. First, we reran the original analyses among participants who were enrolled in the cohort in 1998, 2000, 2002, 2005, 2008 and 2011 and followed up with until 2018, excluding those who were newly enrolled in the cohort in 2014 because a short follow-up period may affect the robustness of the results. Second, we translated age into months and estimated the parameters of monthly transition probabilities, which were used to calculate total and frailty-specific LE (robust LE, prefrailty LE and frailty LE) by *elect*. Third, we reran *elect* with the alternative *"MiddleRiemann"* and *"Simpson"* methods for the numerical approximation [17]. Finally, because cancer is a strong factor influencing LE, we used 37 self-reported health deficits and excluded cancer from the list in Additional file 2 to construct another frailty index, which was used to estimate total and frailty-specific LE.

## Results

The baseline characteristics of the participants are provided in Additional file 5: Table S1. The average age of the participants was 88.45 years (standard deviation, SD: 9.91), and 40.8% were male.

As shown in Additional file 6: Fig. S1, during the follow-up period, the proportions of robust participants who remained robust or transitioned into pre-frail, frail and death statuses were 24.64%, 39.80%, 17.73% and 17.83%, respectively, and the related factors are shown in Additional file 7: Fig. S1. The proportions of pre-frail participants who remained pre-frail or transitioned into robust, frail and death statuses were 34.39%, 10.27%, 28.14% and 27.27%, respectively, and the related factors are shown in Additional file 7: Fig. S2. The proportions of frail participants who remained frail or transitioned into robust, pre-frail and death statuses were 30.69%, 1.68%, 9.38% and 58.24%, respectively, and the related factors can be found in Additional file 7: Fig. S3.

As shown in Table 1, the total LE at age 65 years among older adults in China was 14.74 years (95% CI: 14.52–14.94), of which 4.18 years (95% CI: 4.05–4.30) were robust, 7.46 years (95% CI: 7.31–7.61) pre-frail and 3.10 years (95% CI: 3.01–3.20) frail, respectively. Robust LE varied among those starting at a frail state at age 65 years. Robust participants had a robust LE of 6.10 years (95% CI: 5.93–6.22), which was 40.24% of the total LE (15.16 years). Pre-frail participants had a robust LE of 2.73 years (95% CI: 2.62–2.84), which was 18.65% of total LE (14.64 years). Frail participants had a robust LE of 2.23 years (95% CI: 2.12–2.39), which was 17.19% of the total LE (12.97 years).

**Table 1:** Total and specific state life expectancy by frail states at age 65 years

Frail states at age 65 years	Specific state life expectancy (years)			Total LE	% Robust LE
	**Robust LE**	**Pre-frail LE**	**Frail LE**		
Robust	6.10[5.93–6.22]	6.17[6.01–6.31]	2.89[2.79–2.99]	15.16[15.01–15.25]	40.24
Pre-frail	2.73[2.62–2.84]	8.86[8.67–9.03]	3.05[2.95–3.15]	14.64[14.34–14.93]	18.65
Frail	2.23[2.12–2.39]	5.65[5.44–5.89]	5.09[4.92–5.25]	12.97[12.56–13.37]	17.19
Average	4.18[4.05–4.30]	7.46[7.31–7.61]	3.10[3.01–3.20]	14.74[14.52–14.94]	28.36

Women had a higher total LE (15.60 years, 95% CI: 15.35–15.83) but a lower robust LE (3.58 years, 95% CI: 3.43–3.73) than men (14.16 years, 95% CI: 13.90–14.38; 4.71 years, 95% CI: 4.56–4.88). Total LE was not significantly different among participants with different educational attainment, but participants with more educational attainment had a higher robust LE. Married participants had a higher total LE (15.16 years, 95% CI: 14.93–15.41) and robust LE (4.41 years, 95% CI: 4.27–4.56) than unmarried participants (total LE: 14.03 years, 95% CI: 13.74–14.30; robust LE: 3.64 years, 95% CI: 3.47–3.81) (Table 2).

**Table 2:** Total and specific state life expectancy by demographic characteristics, behaviours, psychosocial factors, and regions

		**Specific state life expectancy (years)**			**Total LE**	**% Robust LE**
		**Robust LE**	**Pre-frail LE**	**Frail LE**		
**Sex**	Male	4.71[4.56–4.88]	7.17[6.96–7.38]	2.28[2.18–2.38]	14.16[13.90–14.38]	33.26
	Female	3.58[3.43–3.73]	7.89[7.68–8.09]	4.13[3.99–4.27]	15.60[15.35–15.83]	22.95
**Years of schooling**	0	3.51[3.37–3.64]	7.39[7.21–7.61]	3.66[3.53–3.79]	14.56[14.31–14.79]	24.11
	1–5	4.21[4.09–4.34]	7.61[7.45–7.78]	2.86[2.76–2.96]	14.68[14.47–14.89]	28.68
	6–11	5.04[4.83–5.23]	7.64[7.40–7.88]	2.18[2.06–2.30]	14.86[14.57–15.14]	33.91
	> = 12	5.96[5.62–6.32]	7.48[7.13–7.87]	1.62[1.48–1.76]	15.06[14.65–15.50]	39.58
**Marital status**	Unmarried	3.64[3.47–3.81]	7.10[6.88–7.34]	3.29[3.13–3.43]	14.03[13.74–14.30]	25.94
	Married	4.41[4.27–4.56]	7.76[7.57–7.93]	2.99[2.88–3.11]	15.16[14.93–15.41]	29.09
**Physical activity**	No	4.02[3.89–4.16]	7.21[7.05–7.39]	3.13[3.02–3.24]	14.36[14.13–14.58]	27.99
	Yes	4.41[4.23–4.59]	7.94[7.71–8.19]	3.06[2.92–3.21]	15.41[15.13–15.69]	28.62
**Smoking**	No	4.00[3.88–4.16]	7.66[7.50–7.83]	3.33[3.22–3.44]	14.99[14.77–15.21]	26.68
	Yes	4.45[4.27–4.68]	7.02[6.77–7.27]	2.50[2.36–2.65]	13.97[13.69–14.28]	31.85
**Drinking**	No	3.97[3.84–4.10]	7.57[7.40–7.74]	3.26[3.14–3.37]	14.80[14.53–14.96]	26.82
	Yes	4.76[4.56–4.98]	7.17[6.92–7.44]	2.70[2.54–2.84]	14.63[14.31–14.94]	32.54
**Daily fruit intake**	Irregularly	4.12[3.99–4.24]	7.41[7.24–7.59]	3.11[3.01–3.22]	14.64[14.42–14.85]	28.14
	Regularly	4.48[4.22–4.77]	7.83[7.51–8.18]	2.97[2.79–3.15]	15.28[14.91–15.69]	29.32
**Daily vegetable intake**	Irregularly	4.13[3.96–4.31]	7.33[7.11–7.57]	3.16[3.02–3.29]	14.62[14.35–14.89]	28.25
	Regularly	4.17[4.04–4.32]	7.54[7.38–7.71]	3.06[2.95–3.17]	14.77[14.54–14.99]	28.23
**Occupation**	PTM	4.80[4.55–5.08]	7.82[7.52–8.15]	2.35[2.19–2.52]	14.97[14.61–15.34]	32.06
	ISWH	4.40[4.25–4.54]	7.66[7.48–7.86]	2.76[2.64–2.87]	14.82[14.58–15.07]	29.69
	AFAF	4.03[3.91–4.16]	7.41[7.26–7.58]	3.21[3.11–3.32]	14.65[14.44–14.86]	27.51
	Other	3.68[3.50–3.88]	7.10[6.86–7.35]	3.71[3.54–3.86]	14.49[14.18–14.78]	25.40
**Social participation**	Low	3.36[3.18–3.53]	6.55[6.33–6.79]	3.07[2.91–3.21]	12.98[12.69–13.24]	25.89
	High	4.39[4.26–4.53]	7.82[7.66–7.99]	3.22[3.11–3.33]	15.43[15.20–15.66]	28.45
**Region**	East China	4.04[3.89–4.18]	7.63[7.44–7.84]	3.07[2.96–3.18]	14.74[14.51–14.98]	27.41
	Central China	4.11[4.01–4.24]	7.50[7.36–7.68]	3.13[3.02–3.13]	14.74[14.53–14.97]	27.88
	Northeast China	4.22[4.13–4.39]	7.38[7.22–7.55]	3.13[3.01–3.24]	14.73[14.51–14.93]	28.65
	West China	4.33[4.18–4.57]	7.26[7.09–7.51]	3.15[3.03–3.28]	14.74[14.46–14.99]	29.38

Regarding health behaviours (Table 2), smokers had a lower total LE (13.97 years, 95% CI: 13.69–14.28) and higher robust LE (4.45 years, 95% CI: 4.27–4.68) than nonsmokers (total LE: 14.99 years, 95% CI: 14.77–15.21; robust LE: 4.00 years, 95% CI: 3.88–4.16). Participants regularly engaging in physical activity had a higher total LE (15.41 years, 95% CI: 15.13–15.69) and robust LE (4.41 years, 95% CI: 4.23–4.59) than their counterparts (total LE: 14.36 years, 95% CI: 14.13–14.58; robust LE: 4.02 years, 95% CI: 3.89–4.16). Total LE was not significantly different among drinkers (14.63 years, 95% CI: 14.31–14.94) and nondrinkers (14.80 years, 95% CI: 14.53–14.96), while the former had a higher robust LE (4.76 years, 95% CI: 4.56–4.98) than the latter (3.97 years, 95% CI: 3.84–4.10). Participants regularly consuming fruits daily had a higher total LE (15.28 years, 95% CI: 14.91–15.69) than their counterparts (14.64 years, 95% CI: 14.42–14.85).

Participants with PTM occupations had the highest robust LE (4.80 years, 95% CI: 4.55–5.08), while those with other occupations had the lowest robust LE (3.68 years, 95% CI: 3.50–3.88). The total LE and frailty-specific LE did not vary among the 4 regions in China (Table 2).

Regarding psychosocial factors (Table 2), participants with high social participation had a higher total LE (15.43 years, 95% CI: 15.20–15.66) and robust LE (4.39 years, 95% CI: 4.26–4.53) than their counterparts (total LE: 12.98 years, 95% CI: 12.69–13.24; robust LE: 3.36 years, 95% CI: 3.18–3.53).

The results of the sensitivity analyses are presented in Additional file 8: Fig. S1-Fig. S25. There were slight changes from the results of the main analyses, but they were not different, which indicated that the findings were robust.

## Discussion

Based on nationally representative data, this 20-year longitudinal study firstly confirmed the dynamically reversible property of frailty [2]. Specifically, 10.27% of pre-frail older adults and only 1.68% of frail older adults improved to robust status, while 27.27% of the former and 58.24% of the latter died during the follow-up period. A recent meta-analysis also revealed that one-quarter of pre-frail older adults improved to robust status while only 3% of frail older adults achieved this status; 13.4% of pre-frail older adults and 32.5% of frail older adults at baseline died during the follow-up period of 1–10 years [31]. Compared with previous studies [31], the improvement rates of pre-frailty and frailty were lower among older adults in China, and the mortality rates of pre-frail and frail older adults were higher. In addition, we also found that high social participation facilitated the improvement of pre-frailty (to robust) and prevented the worsening of pre-frailty (to frailty or death).

Regarding the focus of this study, we found that the total LE of older adults at age 65 years in China was 14.74 years, which was lower than the LE of older adults in 10 European countries (16.4 years at age 70 years) [9], 15 European countries (21.4 years at age 65 years) [10], France (18.3 years for women and 14.8 years for men at age 70 years) [11], and Brazil (15.4 years at age 65 years) [12]. On average, older adults in China are expected to be in robust, pre-frail or frail status for 4.18, 7.46 or 3.10 years, respectively. Frail older adults had a total LE reduction of 2.19 years and a reduction of robust LE of 3.87 years compared with robust older adults in China. These findings further confirm the previous findings that frailty can reduce LE [9–12, 32] and the LE of older adults in the robust category [9–12].

Consistent with previous studies [9–12], the Male‒Female Health-Mortality Paradox [33] existed among older adults, meaning that older women had a longer total LE and frail LE, but a lower robust LE than older men. We also found inequalities in total LE and robust LE by socioeconomic status, which may be explained by social, behavioural, and biological factors throughout life [12, 34]. For example, educational attainment in early life is not associated with total LE, but more educational attainment in early life is associated with longer robust LE in later life. Married older adults at 65 years of age had a longer total LE and lower frail LE than unmarried adults. These findings highlight the complex links among socioeconomic status, life chances, occupation, and life expectancy. For example, those with higher educational attainment in early life may have higher health literacy, healthier behaviours, and better working conditions.

Among the 5 health behaviours, individuals performing regular physical activity or consuming fruits daily at the age of 65 years had a longer total LE and robust LE than their counterparts. Smokers and drinkers had a lower total LE but a higher robust LE than their counterparts. Regarding the factors related to transitions of frailty status, we found that smoking and drinking at 65 years of age were not associated with the transition from frailty to death, but smoking and drinking (not significantly) were associated with the transition from a robust status to death, implying that smokers and drinkers may experience sudden death, for example, acute coronary syndrome [35] or accidents. The alcohol paradox must also be noted: zero alcohol consumption in old age is associated with frailty [36]. Nevertheless, we only qualitatively assessed the drinking behaviour of participants at 65 years of age. Given the benefits of healthy behaviours on life expectancy, these findings suggest that it is important for older adults’ health and longevity to promote engagement in healthy behaviours even in later life.

Finally, but most importantly, previous studies have demonstrated that social participation [37, 38] is associated with mortality and frailty. However, to the best of our knowledge, few studies have investigated the association between social participation and life expectancy. Consistent with the results of two previous studies [37, 39], we found that high social participation prolonged not only total LE but also robust LE. Additionally, inequalities in total and frailty-specific life expectancy were not found in economic regions of China, which indicates that frailty and its impact on life expectancy may be a common challenge in China.

Our findings have some important implications for public health. First, given the dynamically reversible property of frailty, which may lead to reductions in total and robust LE, pre-frail older adults have more opportunity to improve to robust status than frail older adults; thus, early screening and psychosocial interventions are helpful for healthy aging. Second, inequalities in total and robust LE exist within socioeconomic statuses, such as sex, marital status, and educational attainment in early life, which emphasizes the importance of the life course perspective of healthy aging. Third, frailty may lead to reductions in total and robust LE, but healthy behaviours and social participation may ease these reductions; thus, interventions aiming to improve healthy behaviours and social participation should be considered.

This study had several strengths. First, we applied the MSLT method to estimate the total and frailty-specific life expectancy. Although several approaches, such as the Cox model and the Sullivan method, exist for calculating life expectancy, they cannot capture the natural course of a disease or condition. The MSLT describes how individuals move through different stages of frailty and can capture the dynamic nature of frailty. Second, this was a national population-based large cohort study involving 36,348 participants who were followed up with for a long period, which ensures the robustness of our findings, as the sensitivity analyses demonstrated. Third, we analysed various factors, including demographic characteristics, behaviours, and psychosocial factors related to transitions in frailty status and life expectancy, which may provide a comprehensive understanding of the impact of frailty on healthy ageing.

Some limitations should also be noted in this study. First, this is an observational study, and the reverse causality between potential factors and life expectancy should be noted. For example, older adults living longer may have a higher level of social participation. Second, loss to follow-up and obtaining mortality information from the next of kin or the primary caregiver may bias the findings. Third, self-reported data on measures of frailty and subpopulation identifiers may not accurately assess true conditions, which may also bias the findings. Fourth, this study included the whole aging population aged 65–122 years, but most (79.78%) were aged ≥ 80 years, accounting for approximately 18.78% of the entire aging population aged ≥ 65 years in China in 2020. The prevalence of frailty is positively associated with age, and this study may overestimate the impact of frailty on healthy aging in China. Finally, individual characteristics, health behaviours and environments and their interactions may affect frailty prevalence and life expectancy. However, on account of the limitations of the statistical analysis method, we cannot clarify the effects of these interactions on life expectancy, which is worthy of future research.

## Conclusions

Despite the limitations discussed above, our findings confirm that frailty may lead to a reduction in total and robust LE in older adults in China. In addition to finding inequalities in total and robust LE by socioeconomic status, our findings also highlight that healthy behaviours and social participation may ease frailty-related reductions in total and robust LE. Our findings imply that national life-course strategies aimed at frailty screening and psychosocial and behavioural interventions should be important for health aging in China.

## Supplementary Information

**Additional file 1.** Flowchart of participants.

**Additional file 2.** List of items included in the frailty index.

**Additional file 3.** Methods to measure social participation and classification of occupational types.

**Additional file 4.** Estimation methods of life expectancy.

**Additional file 5.** Baseline characteristics of participants.

**Additional file 6: Fig. S1.** Transitions of frail states.

**Additional file 7:   Fig. S1.** Related factors of transitions of robust. **Fig. S2.** Related factors of transitions of pre-frailty. **Fig. S3.** Related factors of transitions of frailty.

**Additional file 8:** Sensitivity analysis results. **Fig. S1.** Life expectancies according to starting state at age 65 years. (Results of monthly transitions). **Fig. S2.** Life expectancies by sex, marital status and years of schooling. (Results of monthly transitions). **Fig. S3.** Life expectancies by behaviours (Results of monthly transitions). **Fig. S4.** Life expectancies by occupations and regions. (Results of monthly transitions). **Fig. S5.** Life expectancies by social participation (Results of monthly transitions). **Fig. S6.** Life expectancies according to starting state at age 65 years (Results of 98-14 cohort). Fig. S7- Life expectancies by sex, marital status and years of schooling (Results of 98-14 cohort). **Fig. S8.** Life expectancies by behaviors (Results of 98-14 cohort). **Fig. S9.** Life expectancies by occupations and regions (Results of 98-14 cohort). **Fig. S10.** Life expectancies by social participation (Results of 98-14 cohort). **Fig. S11.** Life expectancies according to starting state at age 65 years (Results of MiddleRiemann estimation method). **Fig. S12.** Life expectancies by sex, marital status and years of schooling (Results of MiddleRiemann estimation method). **Fig. S13.** Life expectancies by behaviors (Results of MiddleRiemann estimation method). **Fig. S14.** Life expectancies by occupations and regions (Results of MiddleRiemann estimation method). **Fig. S15.** Life expectancies by social participation (Results of MiddleRiemann estimation method). **Fig. S16.** Life expectancies according to starting state at age 65 years (Results of Simpson estimation method). **Fig. S17.** Life expectancies by sex, marital status and years of schooling (Results of Simpson estimation method). **Fig. S18.** Life expectancies by behaviours (Results of Simpson estimation method). **Fig. S19.** Life expectancies by occupations and regions (Results of Simpson estimation method). **Fig. S20.** Life expectancies by social participation (Results of Simpson estimation method). **Fig. S21.** Life expectancies according to starting state at age 65 years (Results of frailty index excluding cancers). **Fig. S22.** Life expectancies by sex, marital status and years of schooling (Results of frailty index excluding cancers). **Fig. S23.** Life expectancies by behaviours (Results of frailty index excluding cancers). **Fig. S24.** Life expectancies by occupations and regions (Results of frailty index excluding cancers). **Fig. S25.** Life expectancies by social participation (Results of frailty index excluding cancers).

## Data Availability

Data are available to researchers on request for purposes of reproducing the results or replicating the procedure by directly contacting the corresponding author.

## Abbreviations

LE: Life expectancy
HALE: Healthy life expectancy
MSLT: Multistate life table
FI: Frailty index
HRs: Hazard ratios
CI: Confidence interval
ADL: Activities of daily living
IADL: Instrumental activities of daily living
SD: Standard deviation
