# Supplementary material for: Life expectancy among older adults with or without frailty in China: multistate modelling of a national longitudinal cohort study
Source: BMC Med. 2023 Mar 16;21:101. doi: 10.1186/s12916-023-02825-7 (PMC10021933; doi:10.1186/s12916-023-02825-7)
Supplement: Supplementary file 1 — Additional file 1. Flowchart of participants. [file 12916_2023_2825_MOESM1_ESM.docx]

**Additional file 1**

**1998**: 9,093

Died: 3,356

Followed survivors: 4,831

Newly: 6,214

**2000**: 11,045

Died: 3,307

Followed survivors: 6,224

Newly: 9,590

**2002**: 15,814

Died: 5,800

Followed survivors: 8,031

Newly: 7,067

**2005**: 15,098

Died: 5,061

Followed survivors: 7,175

Newly:8,840

**2008**: 16,015

Died: 5,379

Followed survivors: 7,890

Newly: 1,276

**2011**: 9,166

Died: 2,715

Followed survivors: 5,669

Newly: 1,125

**2014**: 6,794

Died: 2,092

**2018**：

Total: 43,205

Excluded for only in one wave: 6802

Excluded for missing age or follow-time: 55

**Fig. S1.** **Flowchart of participants from 1998-2018 in CLHLS**
