# Supplementary material for: Life expectancy among older adults with or without frailty in China: multistate modelling of a national longitudinal cohort study
Source: BMC Med. 2023 Mar 16;21:101. doi: 10.1186/s12916-023-02825-7 (PMC10021933; doi:10.1186/s12916-023-02825-7)
Supplement: Supplementary file 2 — Additional file 2. List of items included in the frailty index. [file 12916_2023_2825_MOESM2_ESM.docx]

**Additional file 2**

**Table S1. List of items included in the frailty index**

|  | **Variables** | **Values** |
| --- | --- | --- |
| **1** | Bathing | without assistance=0; assistance with 1 body part=0.5; assistance with >1 body part=1 |
| **2** | Dressing | without assistance=0; some assistance=0.5; unable to dress without assistance=1 |
| **3** | Toileting | without assistance=0; some assistance=0.5; unable to use toilet without assistance=1 |
| **4** | Indoor activities | without assistance=0; some assistance=0.5; a lot of assistance=1 |
| **5** | Continence | able=0; occasional incontinence=0.5; frequent incontinence=1 |
| **6** | Feeding | without assistance=0; some assistance=0.5; a lot of assistance=1 |
| **7** | Hand behind neck | right hand=0.5; left hand=0.5; both hands=0; neither hand=1 |
| **8** | Hand behind lower back | right hand=0.5; left hand=0.5; both hands=0; neither hand=1 |
| **9** | Able to stand up from sitting | yes, without using hands=0; yes, using hands=0.5; no=1 |
| **10** | Able to pick up a book from the floor | yes, standing=0; yes, sitting=0.5; no=1 |
| **11** | Able to use chopsticks to eat | yes=0; no=1 |
| **12** | Number of steps used to turn around a 360 degree turn without help | ≤4=0; >4=0.5; cannot turn around=1 |
| **13** | Visual function | can see and distinguish the break in the circle=0; can see but not distinguish the break in the circle=0.33; cannot see=0.67; blind=1 |
| **14** | Hearing ability | can hear without a hearing aid=0; can hear with a hearing aid=0.33; partly deaf, despite using a hearing aid=0.67; deaf=1 |
| **15** | MMSE | 0:9=1; 10:17=0.67; 18:23=0.33; 24:30=0 |
| **16** | Self-reported health | very good=0; good=0.25; average=0.5; bad=0.75; very bad=1 |
| **17** | Feel fearful or anxious | always=1; often=0.75; sometimes=0.5; seldom=0.25; never=0 |
| **18** | Feel useless because of age | always=1; often=0.75; sometimes=0.5; seldom=0.25; never=0 |
| **19** | Look on the bright side of things | always=0; often=0.25; sometimes=0.5; seldom=0.75; never=1 |
| **20** | Keep my belongings neat and clean | always=0; often=0.25; sometimes=0.5; seldom=0.75; never=1 |
| **21** | Make own decisions | always=0; often=0.25; sometimes=0.5; seldom=0.75; never=1 |
| **22** | Cardiac rhythm | regular=0; irregular=1 |
| **23** | Housework at present | almost every day=0; not daily, but at least once a week=0.5; not weekly, but at least once a month=0.5; not monthly, but sometimes=0.5; never=1 |
| **24** | Interviewer rated-­health | surprisingly healthy=0; relatively healthy=0.33; moderately unhealthy=0.67; very unhealthy=1 |
| **25** | Number of times suffering from serious illness in the past two years | 0=0; 1=1; >1=2 |
| **26** | Able to use chopsticks to eat | yes=0; no=1 |
| **27** | Number of natural teeth | 0:5=1; 6:11=0.8; 12:17=0.6; 18:22=0.4; 23:27=0.2; 28:32=0; |
| **28** | Suffering from hypertension | yes=1; no=0 |
| **29** | Suffering from diabetes | yes=1; no=0 |
| **30** | Suffering from heart disease | yes=1; no=0 |
| **31** | Suffering from stroke or cerebrovascular disease | yes=1; no=0 |
| **32** | Suffering from bronchitis, emphysema, pneumonia, asthma | yes=1; no=0 |
| **33** | Suffering from tuberculosis | yes=1; no=0 |
| **34** | Suffering from cataract |  |
| **35** | Suffering from cancer | yes=1; no=0 |
| **36** | Suffering from gastric or duodenal ulcer | yes=1; no=0 |
| **37** | Suffering from Parkinson’s disease | yes=1; no=0 |
| **38** | Suffering from bedsores | yes=1; no=0 |
