# Supplementary material for: Life expectancy among older adults with or without frailty in China: multistate modelling of a national longitudinal cohort study
Source: BMC Med. 2023 Mar 16;21:101. doi: 10.1186/s12916-023-02825-7 (PMC10021933; doi:10.1186/s12916-023-02825-7)
Supplement: Supplementary file 3 — Additional file 3. Methods to measure social participation and classification of occupational types. [file 12916_2023_2825_MOESM3_ESM.docx]

**Additional file 3**

**1) Methods to measure social participation**

Social participation was assessed by asking participants how often they engaged in the following 8 social activities presently: 1) housework (e.g., cooking and caring for children), 2) growing vegetables & engaging in other fieldwork, 3) engaging in garden work, 4) reading newspapers/books, 5) raising domestic animals/pets, 6) playing cards/mahjong, 7) watching TV or listening to the radio, and 8) attending religious activities. The participants responded to each question: almost every day (coded as 4), not every day but at least once in a week (coded as 3), not every week but once in a month (coded as 2), not every month but sometimes (coded as 1), and never (coded as 0). A total social participation score was created by summing the responses of all 8 social activities when participants were newly enrolled in the cohort separately. Social participation was classified into high and low using the median total social participation score.

**2) Classification of occupational types**

Occupational type was classified by asking participants to answer the question “What was your main occupation before age 60?”, with the following choices: ①professional and technical personnel;②governmental, institutional or managerial personnel; ③agriculture, forest, or animal husbandry worker; ④fishery worker; ⑤industrial worker; ⑥commercial or service worker; ⑦military personnel; ⑧housework; and⑨others. We recoded occupational type into 4 types: type 1 (including choices ① and ②, PTM), type 2 (including choices③ and ④, AFAF), type 3 (including choices⑤ and ⑥, ISWH) and type 4 (including choices⑦, ⑧ and ⑨, Other).
