# Supplementary material for: Life expectancy among older adults with or without frailty in China: multistate modelling of a national longitudinal cohort study
Source: BMC Med. 2023 Mar 16;21:101. doi: 10.1186/s12916-023-02825-7 (PMC10021933; doi:10.1186/s12916-023-02825-7)
Supplement: Supplementary file 4 — Additional file 4. Estimation methods of life expectancy. [file 12916_2023_2825_MOESM4_ESM.docx]

**Additional file 4: Estimation methods of life expectancy**

In this study, state-specific life expectancy was estimated based on a time-continuous multi-state model by *elect(1)* and *msm(2)* package of R(3). This method allows users to compute life expectancies for any number of states and is not limited to progressive processes.

A multi-state model including 3 alive state (robust: state 1, pre-frailty: state 2 and frailty: state 3) and a single absorbing state (death: state 4) was defined as shown in **Figure 1**. Given the dynamic nature of frailty, the model permits transitions (shown with arrows) from any alive state to any state (including the same state and the dead state).

Let the finite state space be given by{1,2,…,D} where D is the dead state. Let *Y*_t_ denote the state at age *t* and let *x* denote the time-independent vector with covariate values.

Life expectancy in living state *s* given state *r* at age *t*, for *r*, *s*$\in\left\{ 1,2,...,D-1 \right\},$ is defined by

$e_{rs}\left( t | \boldsymbol{x} \right)=\int_{0}^{\infty} \mathbb{P}\left( Y_{t+u}=s | Y_{t}=r\boldsymbol{,x} \right)du$ (1)

Where ℙ$\left( Y_{t+u}=s | Y_{t}=r\boldsymbol{,x} \right)$ is the transition probability pf being in state *s* at age *t+u*, given starting state *r* at age *t* and covariate values ***x***. Marginal life expectancy in state *s* is irrespective of the initial state at age *t* and is defined by

(2)

$$e_{\cdot s}\left( t | \boldsymbol{x} \right)=\sum_{r\neq4} \mathbb{P}\left( Y_{t}=r | \boldsymbol{x} \right)e_{rs}\left( t | \boldsymbol{x} \right)$$

Where $\mathbb{P}\left( Y_{t}=r | \boldsymbol{x} \right)$ is the probability of being in state *r* at age *t* for r$\in1,2,\ldots,D-1$. Total life expectancy at age *t* is defined as

(3)

$$e\left( t | \boldsymbol{x} \right)=\sum_{s\neq4} e_{\cdot s}\left( t | \boldsymbol{x} \right)$$

To be able to estimate life expectancy, transition probabilities and the state distribution are estimated using longitudinal data. Using the same notation as above, we assume that data for individual *i* and observation *j* are given by (*y_ij_*, *t_ij_*, *x_i_*), for *i* $\in${1,…,N} and *j*$\in${1,…,*n_i_*}. Transition probabilities are derived from a multi-state model where the hazards are defined by

(4)

$$h_{rs}\left( t_{ij} \right)=exp\left( \beta_{rs}+\xi_{rs}t_{ij}+\gamma_{rs}x_{i} \right)$$

This model is estimated using *msm* package.

The distribution of the state at age *t* is modelled using a multinomial regression model defined by

(5)

$$\mathbb{P}\left( Y_{t}=r | \boldsymbol{x} \right)=\frac{exp\left( \eta_{r}\left( t \right) \right)}{1+\sum_{r\neq D} exp(\eta_{r}\left( t \right))}$$

with $\eta_{r}\left( t \right)=\alpha_{r0}+\alpha_{r1}t+\alpha_{r2}x$

for r∈{1, 2, ..., D−1}. By restricting *α_10_* = *α_11_* = *α_12_* = 0, we make *r*=1 the reference category. This model is estimated in *elect* using the function *multinom* in the package *nnet.*

Life expectancies (1), (2), and (3) can be derived by *elect* package using the parameters in the multi-state model and the multinomial regression model.

**References**

1. van den Hout A, Sum Chan M, Matthews F. Estimation of life expectancies using continuous-time multi-state models. Comput Methods Programs Biomed. 2019;178:11-8.

2. Jackson C. Multi-State Models for Panel Data: The msm Package for R. Journal of Statistical Software. 2011;38(8):1 - 28.

3. Team RC, . R: A language and environment for statistical computing Vienna, Austria: R Foundation for Statistical Computing; 2022 [Available from: <https://www.R-project.org/>.
