# Supplementary material for: Life expectancy among older adults with or without frailty in China: multistate modelling of a national longitudinal cohort study
Source: BMC Med. 2023 Mar 16;21:101. doi: 10.1186/s12916-023-02825-7 (PMC10021933; doi:10.1186/s12916-023-02825-7)
Supplement: Supplementary file 5 — Additional file 5. Baseline characteristics of participants. [file 12916_2023_2825_MOESM5_ESM.docx]

**Additional file 5: Baseline characteristics of participants**

**Table S1. Baseline characteristics of participants, n (%)**

|  | | **Enrolled in 1998** | **Enrolled in 2000** | **Enrolled in 2002** | **Enrolled in 2005** | **Enrolled in 2008** | **Enrolled in 2011** | **Enrolled in 2014** | **Total** |
| --- | --- | --- | --- | --- | --- | --- | --- | --- | --- |
| N | | 8,184 | 5,278 | 8,313 | 5,633 | 7,031 | 1,023 | 886 | 36,348 |
| Age (mean (SD)) | | 90.51 (7.51) | 92.36 (7.70) | 83.19 (12.85) | 88.30 (12.46) | 90.01 (11.34) | 88.58 (12.31) | 88.45 (9.91) | 88.71 (11.21) |
| Sex | Male | 3245 (39.7) | 2237 (42.4) | 3550 (42.7) | 2253 (40.0) | 2761 (39.3) | 419 (41.0) | 14845 (40.8) | 3245 (39.7) |
|  | Female | 4939 (60.3) | 3041 (57.6) | 4763 (57.3) | 3380 (60.0) | 4270 (60.7) | 604 (59.0) | 21503 (59.2) | 4939 (60.3) |
| Years of schooling | 0 | 5567 (68.4) | 3345 (64.0) | 5162 (62.5) | 3713 (66.2) | 4818 (68.7) | 697 (69.2) | 23891 (66.2) | 5567 (68.4) |
|  | 1-5 | 1741 (21.4) | 1261 (24.1) | 1903 (23.0) | 1142 (20.3) | 1263 (18.0) | 191 (19.0) | 7646 (21.2) | 1741 (21.4) |
|  | 6-11 | 626 (7.7) | 461 (8.8) | 934 (11.3) | 578 (10.3) | 785 (11.2) | 109 (10.8) | 3598 (10.0) | 626 (7.7) |
|  | >=12 | 208 (2.6) | 159 (3.0) | 261 (3.2) | 179 (3.2) | 148 (2.1) | 10 (1.0) | 977 (2.7) | 208 (2.6) |
| Marriage status | Unmarried | 6787 (83.0) | 4147 (78.6) | 5061 (60.9) | 4051 (71.9) | 5070 (72.1) | 675 (66.3) | 26390 (72.6) | 6787 (83.0) |
|  | Married | 1394 (17.0) | 1130 (21.4) | 3252 (39.1) | 1582 (28.1) | 1961 (27.9) | 343 (33.7) | 9938 (27.4) | 1394 (17.0) |
| Occupation before retirement | PTM | 512 (6.3) | 356 (6.8) | 675 (8.2) | 348 (6.2) | 330 (4.7) | 23 (2.3) | 2273 (6.3) | 512 (6.3) |
|  | ISWH | 1289 (15.8) | 930 (17.6) | 1202 (14.6) | 898 (16.0) | 755 (10.7) | 57 (5.7) | 5193 (14.4) | 1289 (15.8) |
|  | AFAF | 4639 (56.7) | 2727 (51.7) | 5324 (64.6) | 3588 (63.8) | 5190 (73.8) | 843 (84.6) | 22991 (63.6) | 4639 (56.7) |
|  | Other | 1741 (21.3) | 1261 (23.9) | 1046 (12.7) | 793 (14.1) | 754 (10.7) | 73 (7.3) | 5718 (15.8) | 1741 (21.3) |
| Daily fruit intake | Irregularly | 7025 (85.9) | 4352 (82.5) | 7491 (90.2) | 5015 (89.0) | 6182 (87.9) | 938 (91.8) | 31762 (87.4) | 7025 (85.9) |
|  | Regularly | 1151 (14.1) | 924 (17.5) | 818 (9.8) | 618 (11.0) | 849 (12.1) | 84 (8.2) | 4568 (12.6) | 1151 (14.1) |
| Daily vegetable intake | Irregularly | 2239 (27.4) | 1434 (27.2) | 3860 (46.5) | 2841 (50.4) | 2773 (39.4) | 576 (56.7) | 14141 (38.9) | 2239 (27.4) |
|  | Regularly | 5937 (72.6) | 3843 (72.8) | 4446 (53.5) | 2792 (49.6) | 4258 (60.6) | 440 (43.3) | 22181 (61.1) | 5937 (72.6) |
| Smoking | Yes | 1419 (17.3) | 929 (17.6) | 1694 (20.4) | 1027 (18.2) | 1233 (17.5) | 153 (15.0) | 6564 (18.1) | 1419 (17.3) |
|  | No | 6762 (82.7) | 4349 (82.4) | 6619 (79.6) | 4606 (81.8) | 5798 (82.5) | 866 (85.0) | 29774 (81.9) | 6762 (82.7) |
| Drinking | Yes | 1956 (23.9) | 1090 (20.7) | 1793 (21.6) | 1094 (19.4) | 1287 (18.3) | 153 (15.0) | 7482 (20.6) | 1956 (23.9) |
|  | No | 6221 (76.1) | 4187 (79.3) | 6519 (78.4) | 4539 (80.6) | 5744 (81.7) | 865 (85.0) | 28841 (79.4) | 6221 (76.1) |
| Regularly physical activity | Yes | 2140 (26.2) | 1667 (31.6) | 2540 (30.6) | 1474 (26.2) | 1623 (23.1) | 122 (12.2) | 9686 (26.7) | 2140 (26.2) |
|  | No | 6038 (73.8) | 3610 (68.4) | 5772 (69.4) | 4159 (73.8) | 5408 (76.9) | 877 (87.8) | 26609 (73.3) | 6038 (73.8) |
| Social participation | Low | 4716 (57.8) | 3225 (61.1) | 4187 (50.4) | 3240 (57.5) | 4247 (60.4) | 604 (59.0) | 20698 (57.0) | 4716 (57.8) |
|  | High | 3442 (42.2) | 2053 (38.9) | 4126 (49.6) | 2393 (42.5) | 2784 (39.6) | 419 (41.0) | 15624 (43.0) | 3442 (42.2) |
| Region | East China | 3662 (44.7) | 2213 (41.9) | 3471 (41.8) | 2222 (39.4) | 3187 (45.3) | 513 (50.1) | 15759 (43.4) | 3662 (44.7) |
|  | Central China | 1539 (18.8) | 1122 (21.3) | 1743 (21.0) | 1267 (22.5) | 1825 (26.0) | 472 (46.1) | 8266 (22.7) | 1539 (18.8) |
|  | Northeast China | 518 (6.3) | 582 (11.0) | 805 (9.7) | 533 (9.5) | 405 (5.8) | 0 (0.0) | 2843 (7.8) | 518 (6.3) |
|  | West China | 2465 (30.1) | 1361 (25.8) | 2294 (27.6) | 1611 (28.6) | 1614 (23.0) | 38 (3.7) | 9480 (26.1) | 2465 (30.1) |
