# Supplementary figures and images for: Life expectancy among older adults with or without frailty in China: multistate modelling of a national longitudinal cohort study
Source: BMC Med. 2023 Mar 16;21:101. doi: 10.1186/s12916-023-02825-7 (PMC10021933; doi:10.1186/s12916-023-02825-7)

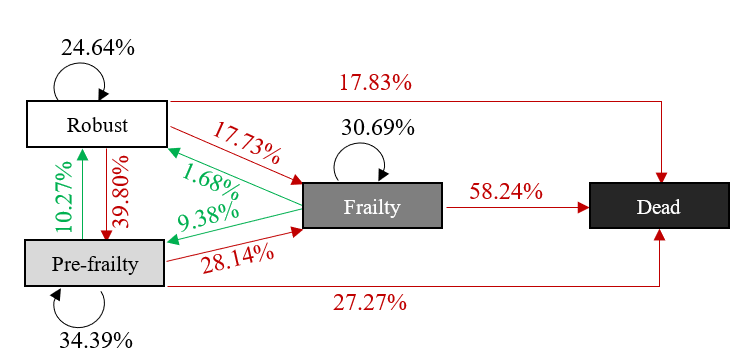
**Additional file 6: Transitions of frail states**

**Fig. S1. transitions of frail states**

Supplement: Supplementary file 6 — Additional file 6: Fig. S1. Transitions of frail states. [file 12916_2023_2825_MOESM6_ESM.docx]
