# Supplementary material for: Life expectancy among older adults with or without frailty in China: multistate modelling of a national longitudinal cohort study
Source: BMC Med. 2023 Mar 16;21:101. doi: 10.1186/s12916-023-02825-7 (PMC10021933; doi:10.1186/s12916-023-02825-7)
Supplement: Supplementary file 7 — Additional file 7: Fig. S1. Related factors of transitions of robust. Fig. S2. Related factors of transitions of pre-frailty. Fig. S3. Related factors of transitions of frailty. [file 12916_2023_2825_MOESM7_ESM.docx]

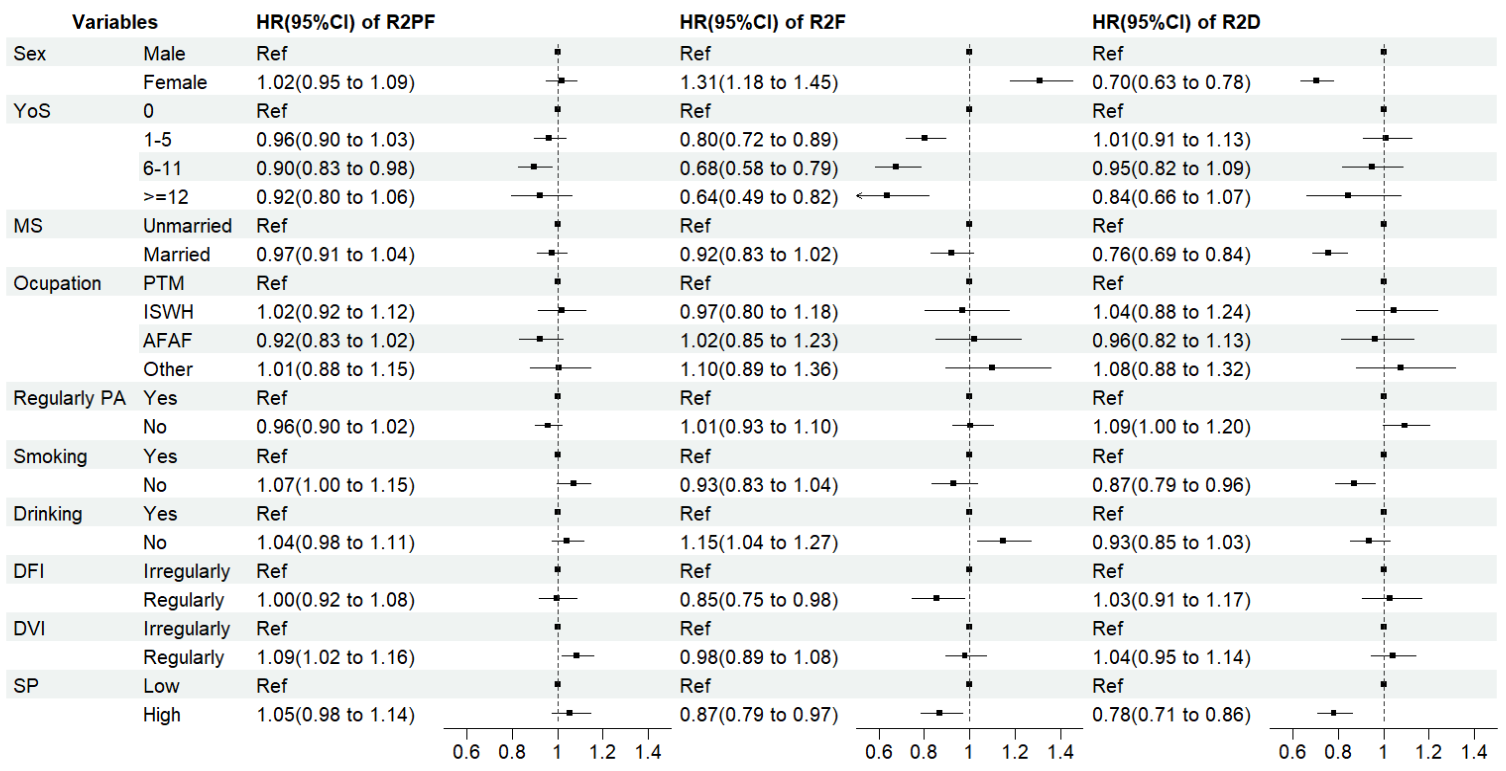
**Additional file 7: Related factors of transitions of frail states**

R2PF: Robust to Pre-frailty; R2F: Robust to Frailty; R2D: Robust to Death; Yos: Years of schooling; MS: Marriage status; Occupation: Occupation before retirement; PA: Physical activity; DFI: Daily fruit intake; DVI: Daily vegetable intake; SP: Social participation

**Fig. S1. Related factors of transitions of robust**


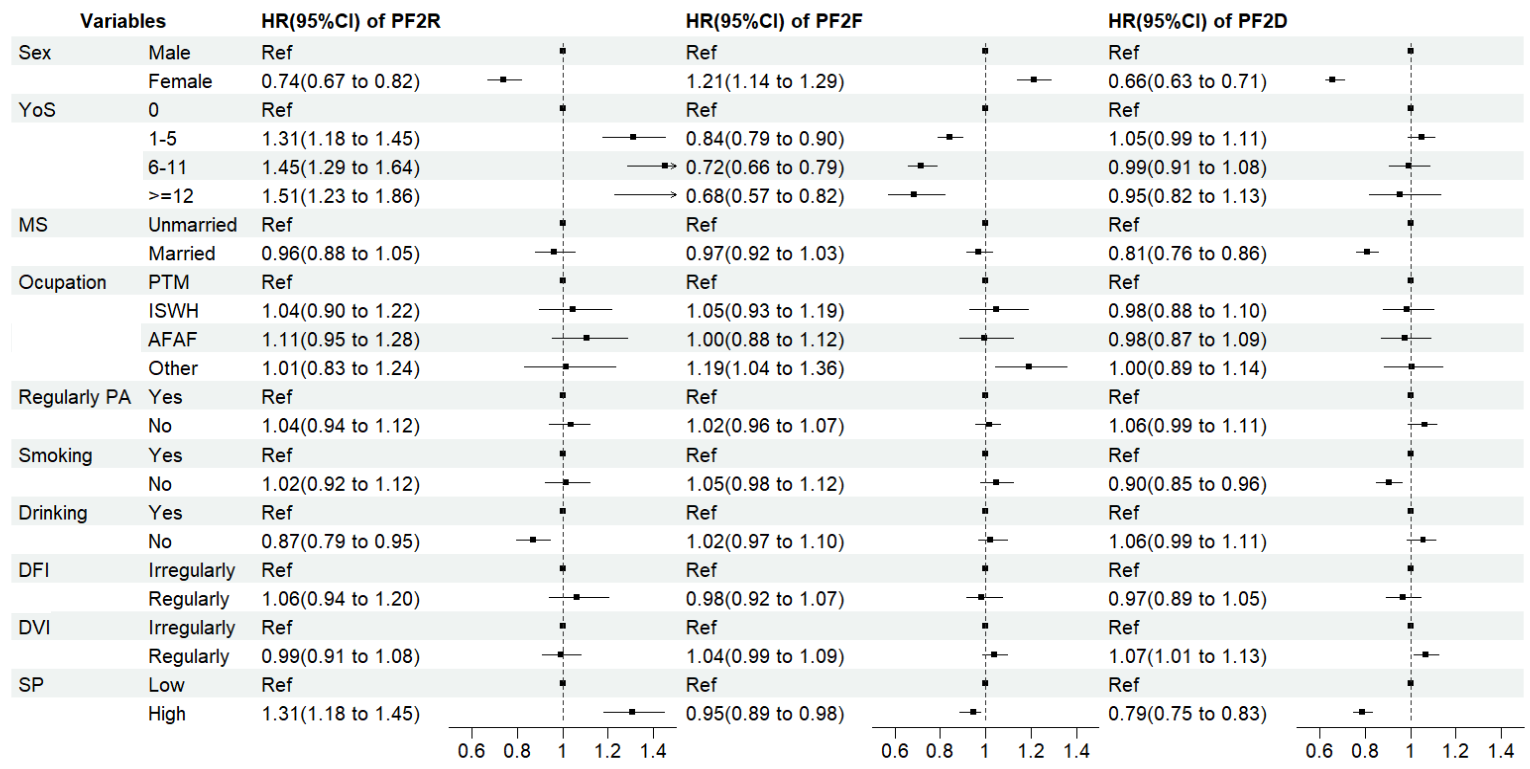


PF2R: Pre-frailty to Robust; PF2F: Pre-frailty to Frailty; PF2D: Pre-frailty to Death; Yos: Years of schooling; MS: Marriage status; Occupation: Occupation before retirement; PA: Physical activity; DFI: Daily fruit intake; DVI: Daily vegetable intake; SP: Social participation

**Fig. S2. Related factors of transitions of pre-frailty**


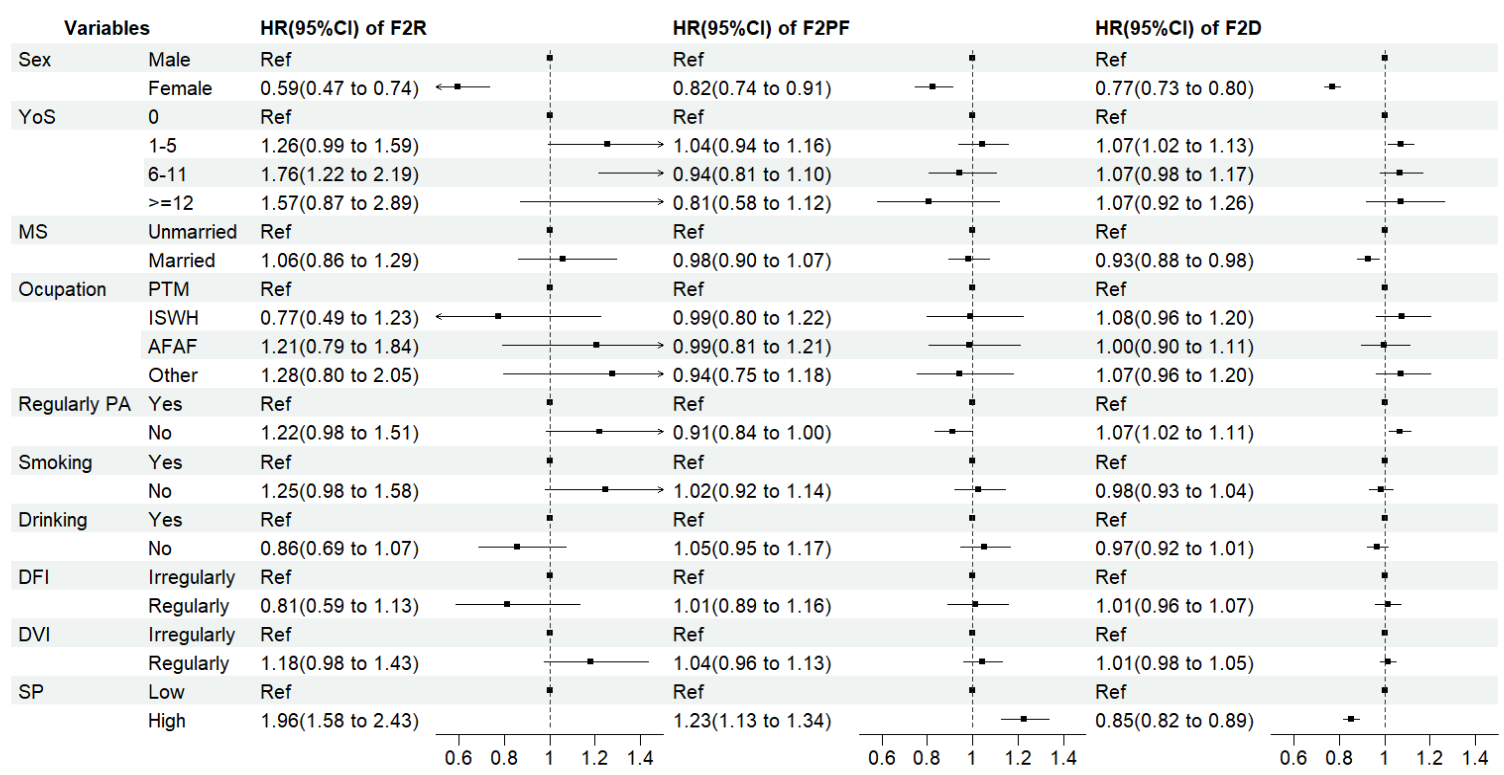


F2R: Frailty to Robust; F2PF: Frailty to Pre-frailty; F2D: Frailty to Death; Yos: Years of schooling; MS: Marriage status; Occupation: Occupation before retirement; PA: Physical activity; DFI: Daily fruit intake; DVI: Daily vegetable intake; SP: Social participation

**Fig. S3. Related factors of transitions of frailty**
