# Supplementary material for: Life expectancy among older adults with or without frailty in China: multistate modelling of a national longitudinal cohort study
Source: BMC Med. 2023 Mar 16;21:101. doi: 10.1186/s12916-023-02825-7 (PMC10021933; doi:10.1186/s12916-023-02825-7)
Supplement: Supplementary file 8 — Additional file 8: Sensitivity analysis results. Fig. S1. Life expectancies according to starting state at age 65 years. (Results of monthly transitions). Fig. S2. Life expectancies by sex, marital status and years of schooling. (Results of monthly transitions). Fig. S3. Life expectancies by behaviours (Results of monthly transitions). Fig. S4. Life expectancies by occupations and regions. (Results of monthly transitions). Fig. S5. Life expectancies by social participation (Results of monthly transitions). Fig. S6. Life expectancies according to starting state at age 65 years (Results of 98-14 cohort). Fig. S7- Life expectancies by sex, marital status and years of schooling (Results of 98-14 cohort). Fig. S8. Life expectancies by behaviors (Results of 98-14 cohort). Fig. S9. Life expectancies by occupations and regions (Results of 98-14 cohort). Fig. S10. Life expectancies by social participation (Results of 98-14 cohort). Fig. S11. Life expectancies according to starting state at age 65 years (Results of MiddleRiemann estimation method). Fig. S12. Life expectancies by sex, marital status and years of schooling (Results of MiddleRiemann estimation method). Fig. S13. Life expectancies by behaviors (Results of MiddleRiemann estimation method). Fig. S14. Life expectancies by occupations and regions (Results of MiddleRiemann estimation method). Fig. S15. Life expectancies by social participation (Results of MiddleRiemann estimation method). Fig. S16. Life expectancies according to starting state at age 65 years (Results of Simpson estimation method). Fig. S17. Life expectancies by sex, marital status and years of schooling (Results of Simpson estimation method). Fig. S18. Life expectancies by behaviours (Results of Simpson estimation method). Fig. S19. Life expectancies by occupations and regions (Results of Simpson estimation method). Fig. S20. Life expectancies by social participation (Results of Simpson estimation method). Fig. S21. Life expectancies according to sta [file 12916_2023_2825_MOESM8_ESM.docx]

**Additional file 8: Sensitivity analysis results**


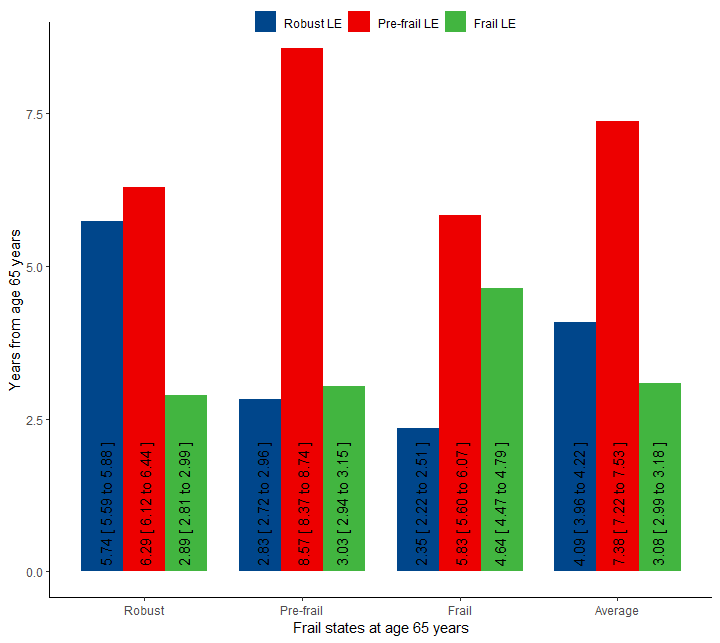


**Fig. S1. Life expectancies according to starting state at age 65 years**

**(Results of monthly transitions)**


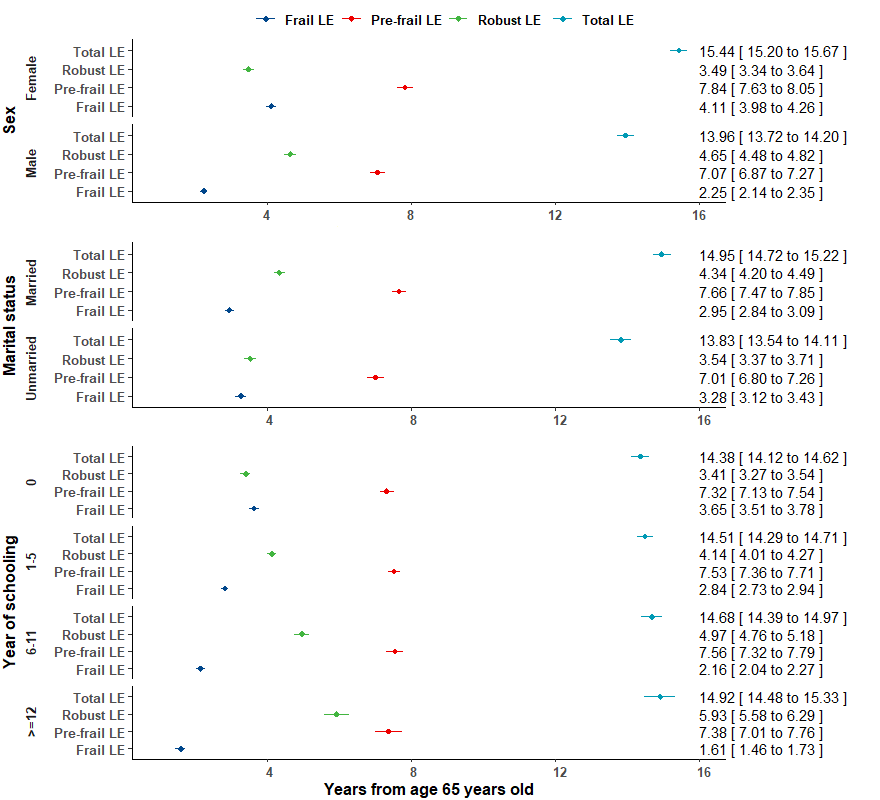


**Fig. S2. Life expectancies by sex, marital status and years of schooling**

**(Results of monthly transitions)**


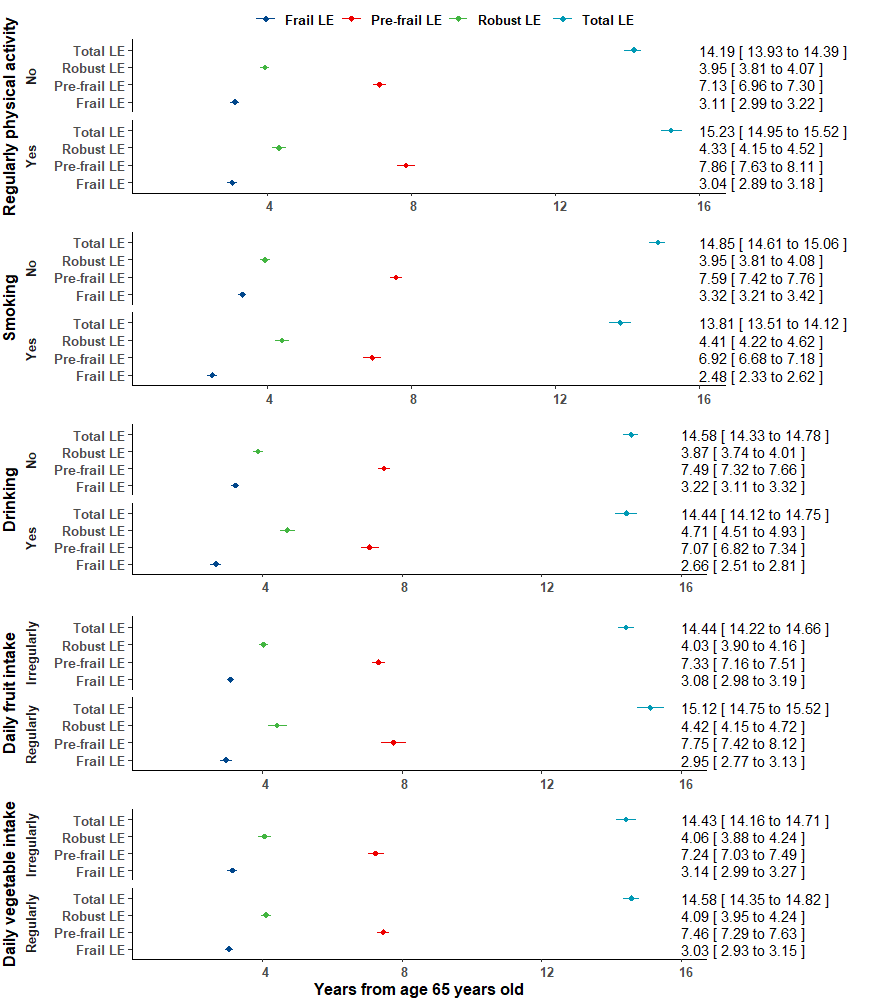


**Fig. S3. Life expectancies by behaviours (Results of monthly transitions)**


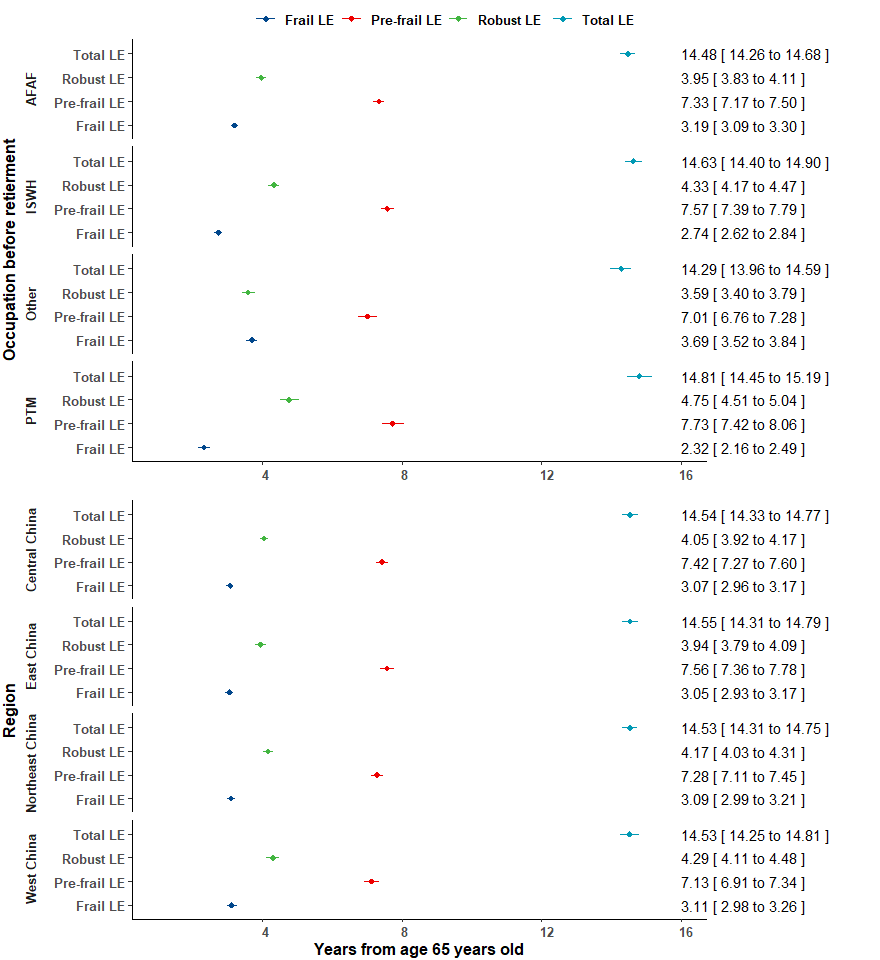


**Fig. S4. Life expectancies by occupations and regions**

**(Results of monthly transitions)**


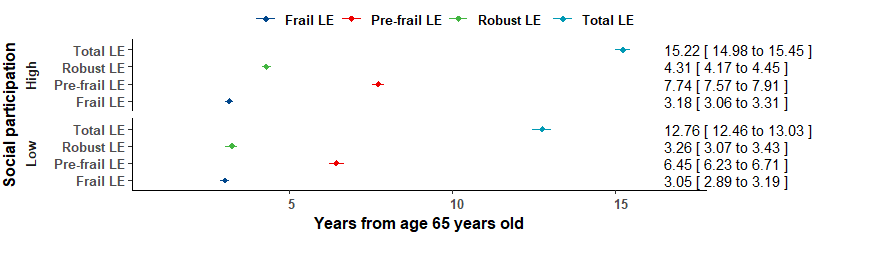


**Fig. S5. Life expectancies by social participation (Results of monthly transitions)**


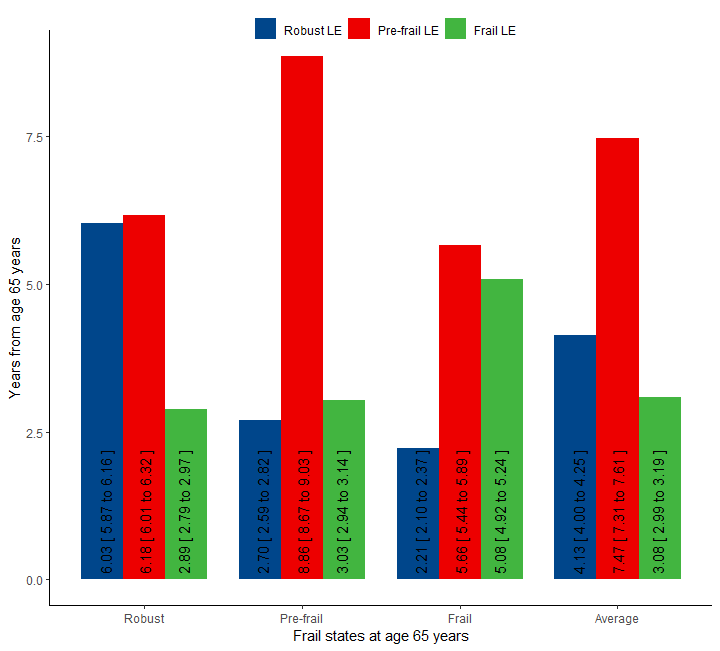


**Fig. S6. Life expectancies according to starting state at age 65 years**

**(Results of 98-14 cohort)**


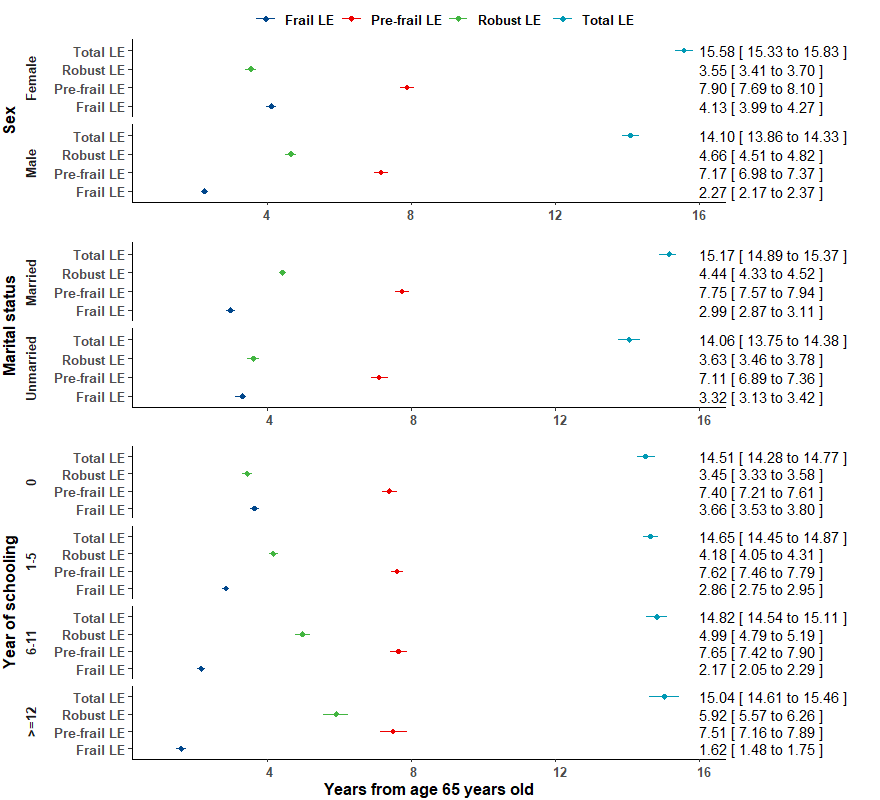


**Fig. S7. Life expectancies by sex, marital status and years of schooling**

**(Results of 98-14 cohort)**


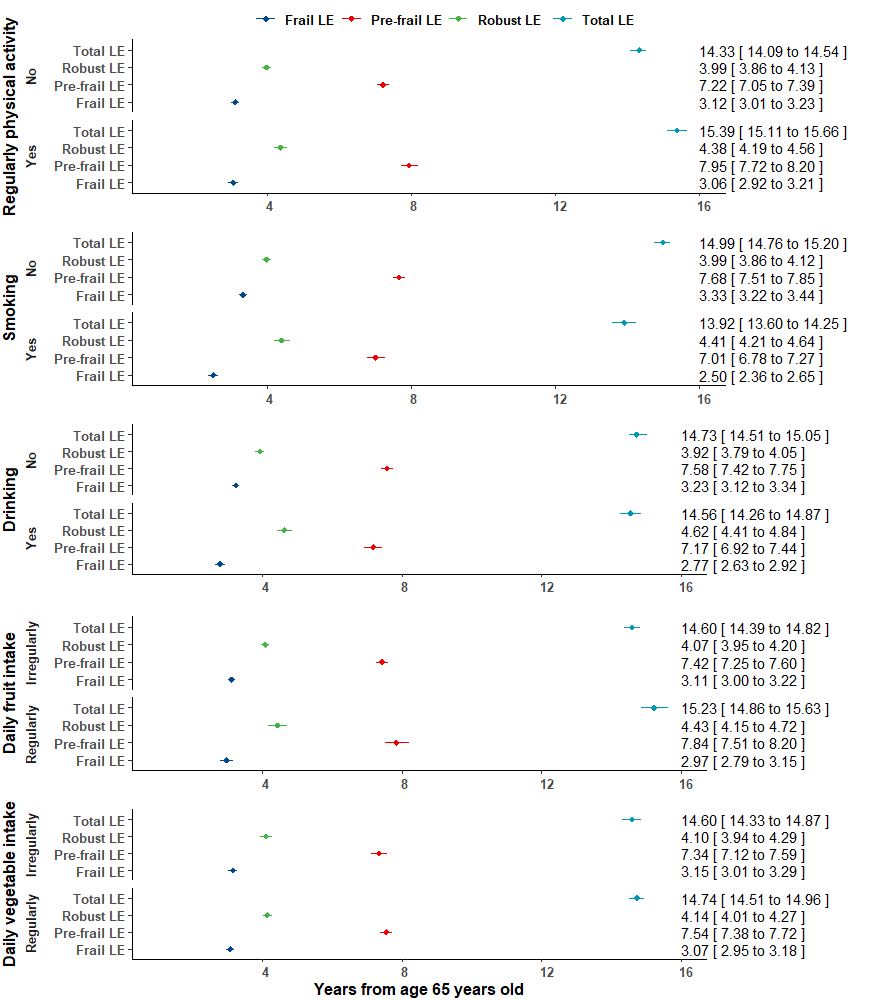


**Fig. S8. Life expectancies by behaviors (Results of 98-14 cohort)**


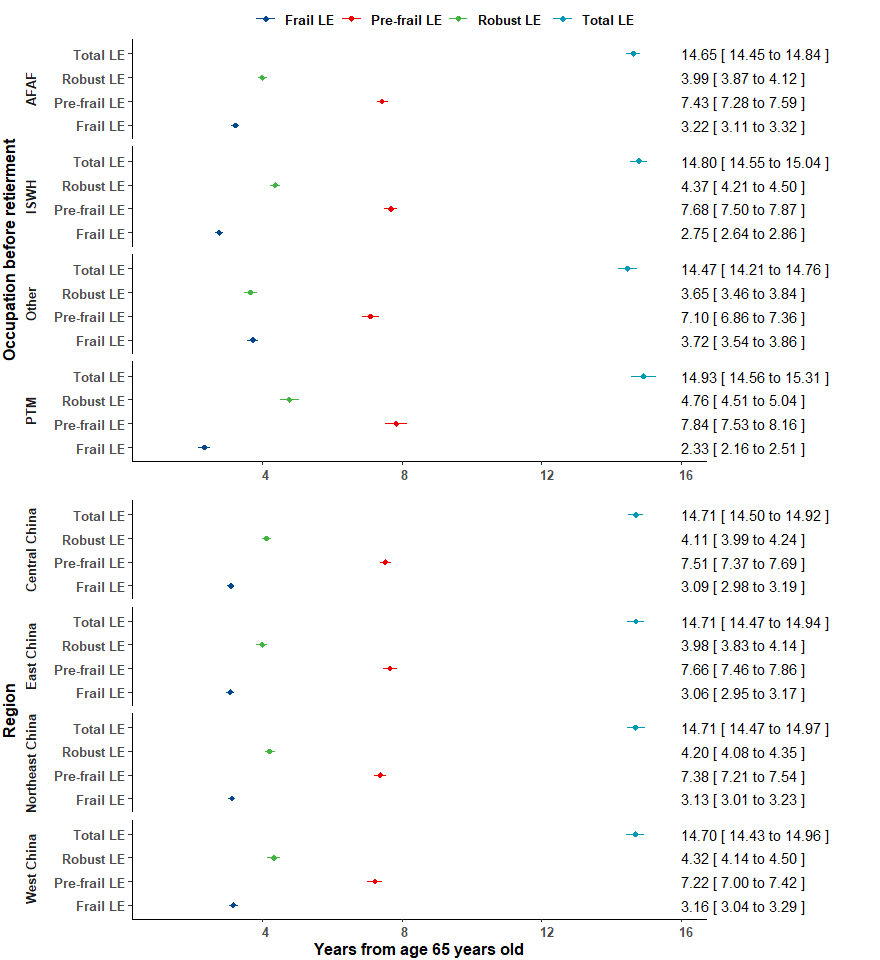


**Fig. S9. Life expectancies by occupations and regions (Results of 98-14 cohort)**


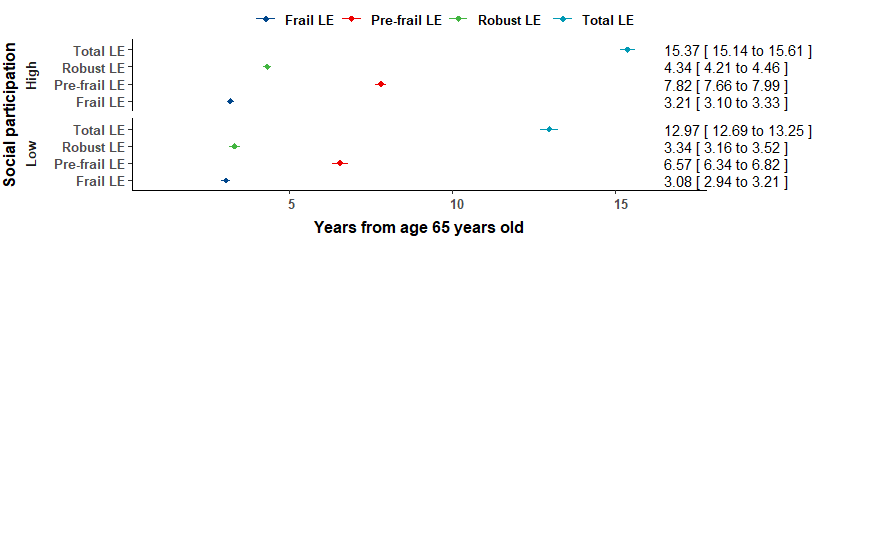


**Fig. S10. Life expectancies by social participation (Results of 98-14 cohort)**


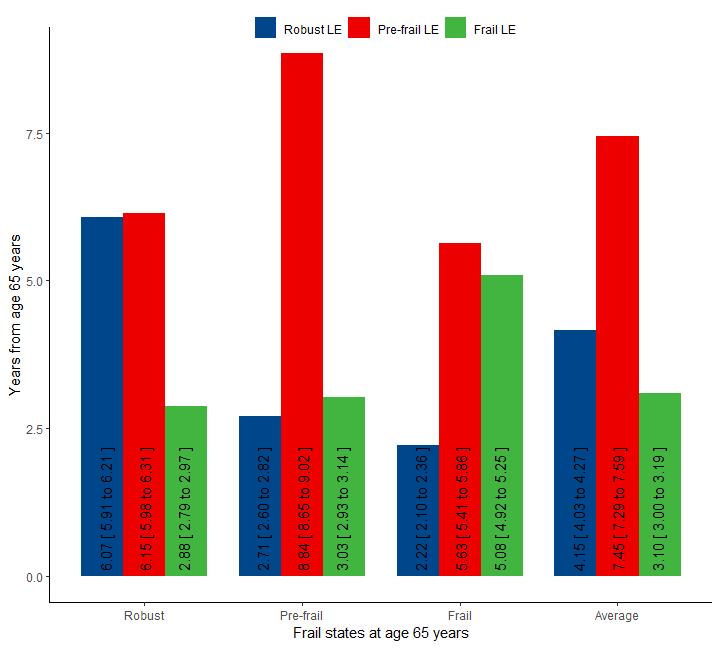


**Fig. S11. Life expectancies according to starting state at age 65 years**

**(Results of** ***MiddleRiemann* estimation method)**


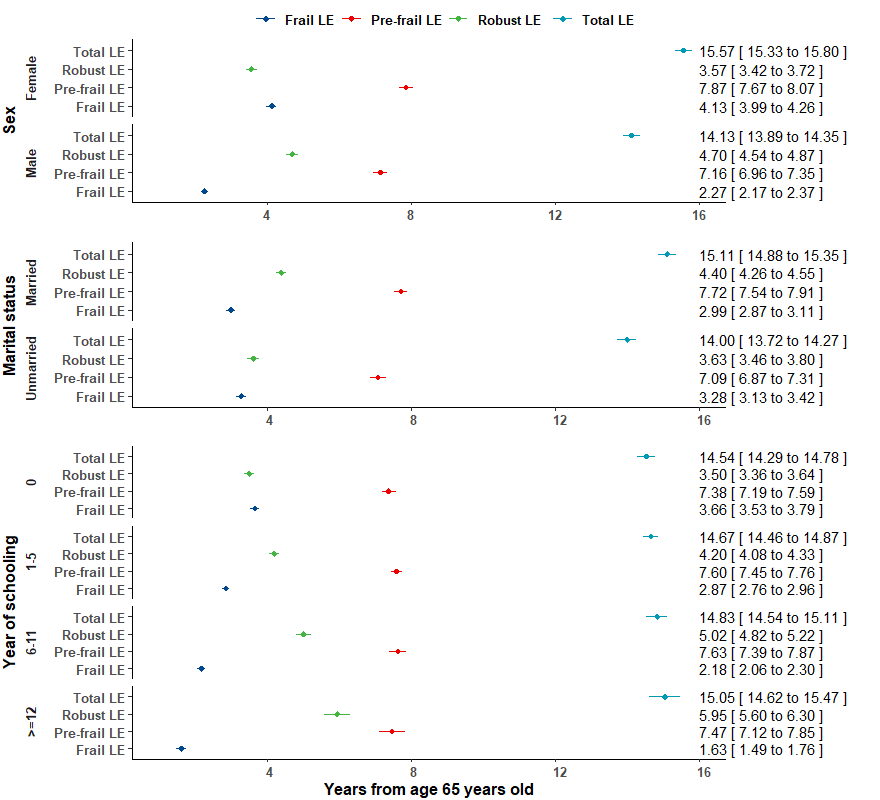


**Fig. S12. Life expectancies by sex, marital status and years of schooling**

**(Results of** ***MiddleRiemann* estimation method)**


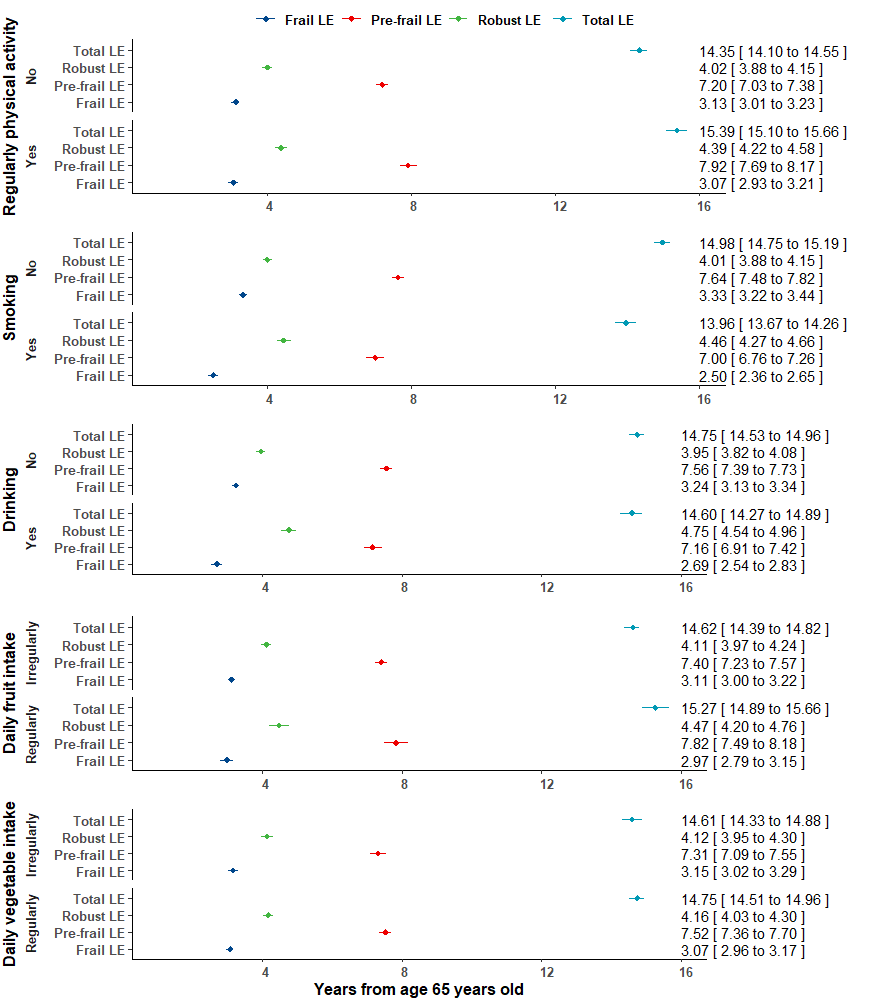


**Fig. S13. Life expectancies by behaviors**

**(Results of** ***MiddleRiemann* estimation method)**


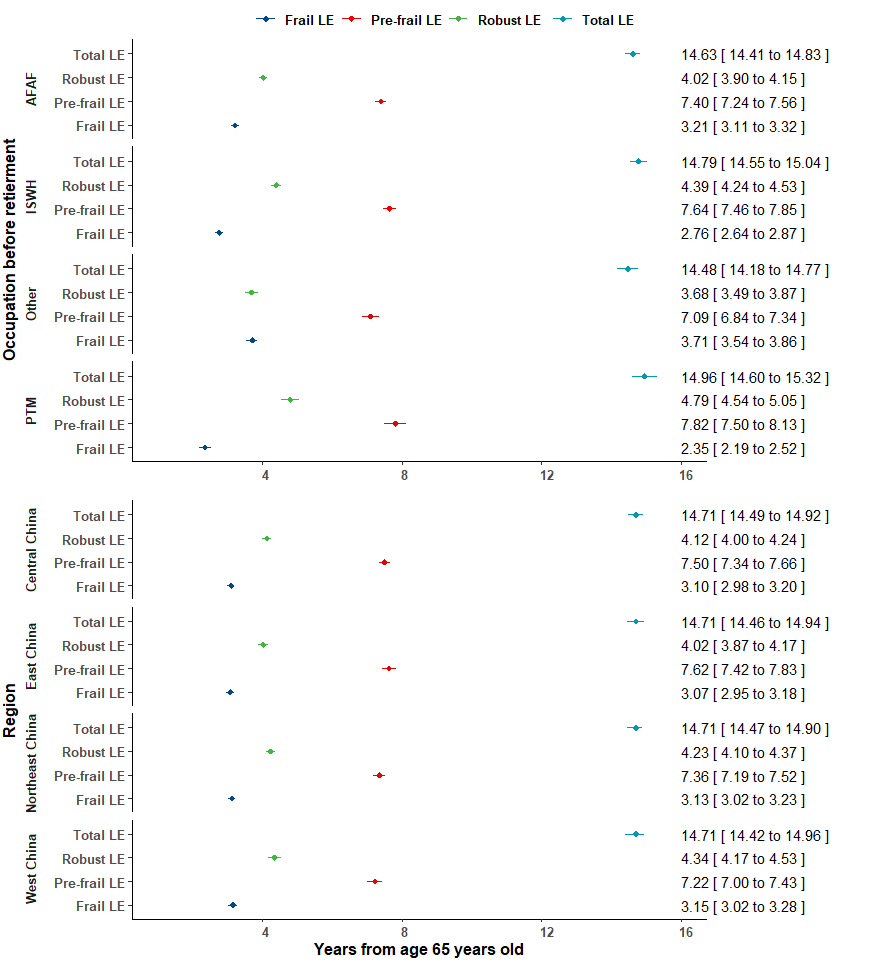


**Fig. S14. Life expectancies by occupations and regions**

**(Results of** ***MiddleRiemann* estimation method)**


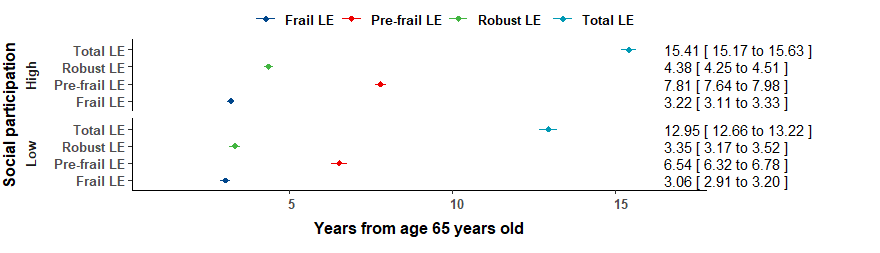


**Fig. S15. Life expectancies by social participation**

**(Results of** ***MiddleRiemann* estimation method)**


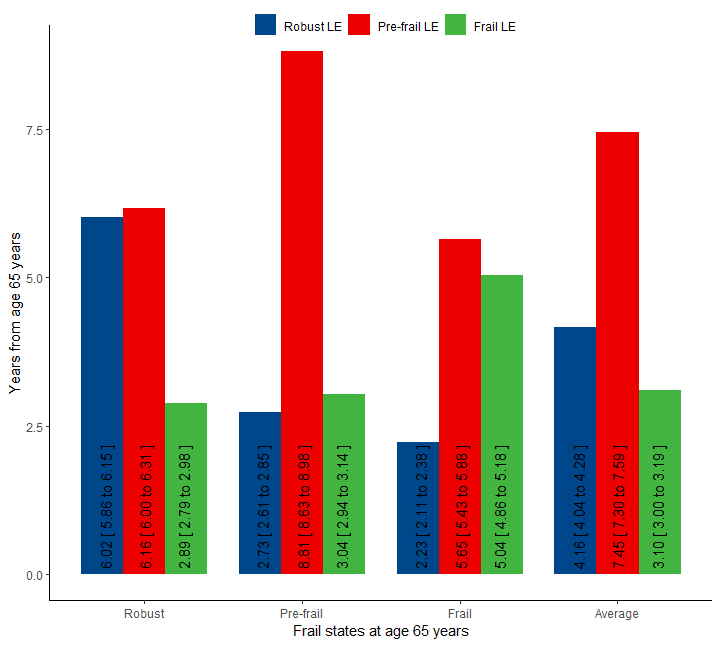


**Fig. S16. Life expectancies according to starting state at age 65 years**

**(Results of** ***Simpson*** **estimation method)**


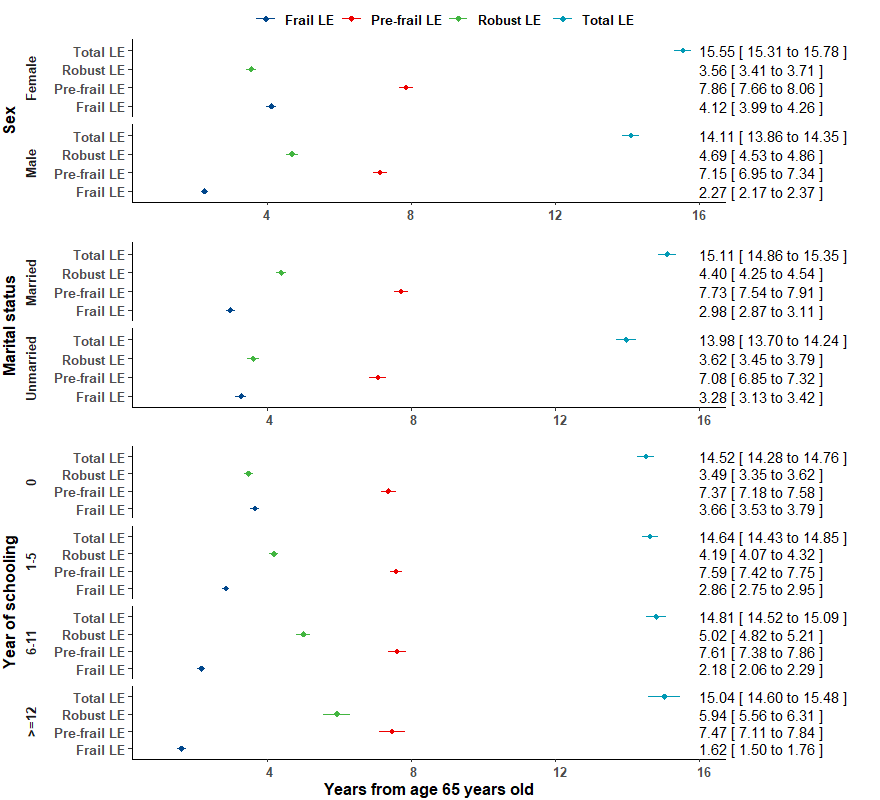


**Fig. S17. Life expectancies by sex, marital status and years of schooling**

**(Results of** ***Simpson*** **estimation method)**


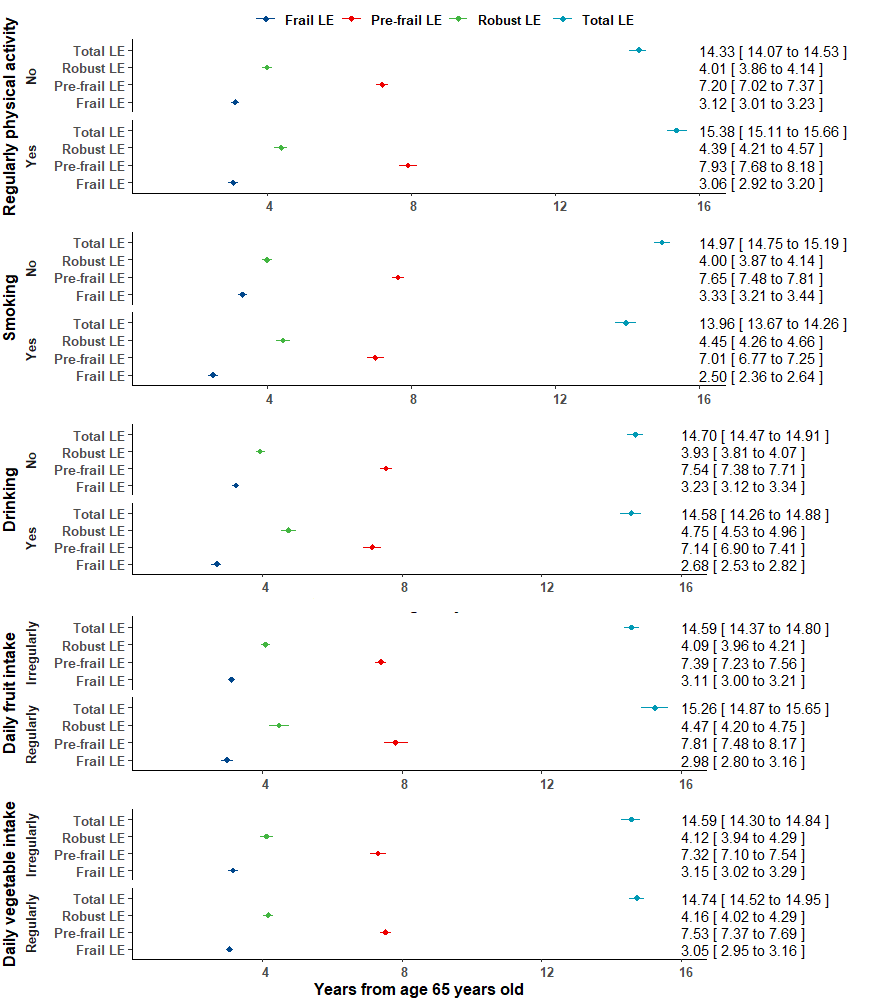


**Fig. S18. Life expectancies by behaviours**

**(Results of** ***Simpson*** **estimation method)**


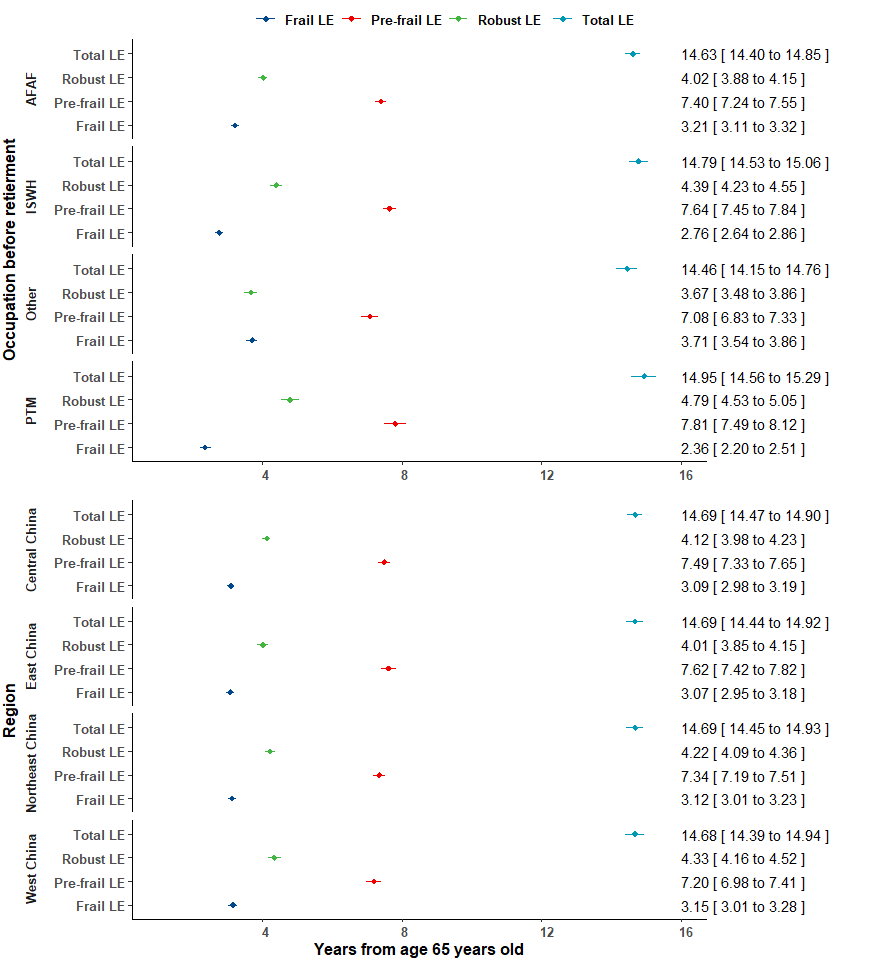


**Fig. S19. Life expectancies by occupations and regions**

**(Results of** ***Simpson*** **estimation method)**


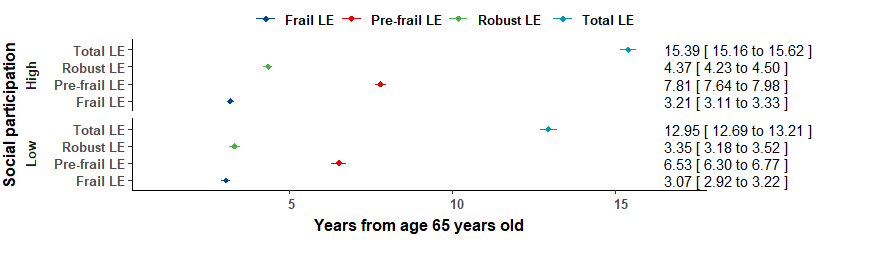
 **Fig. S20. Life expectancies by social participation**

**(Results of** ***Simpson*** **estimation method)**


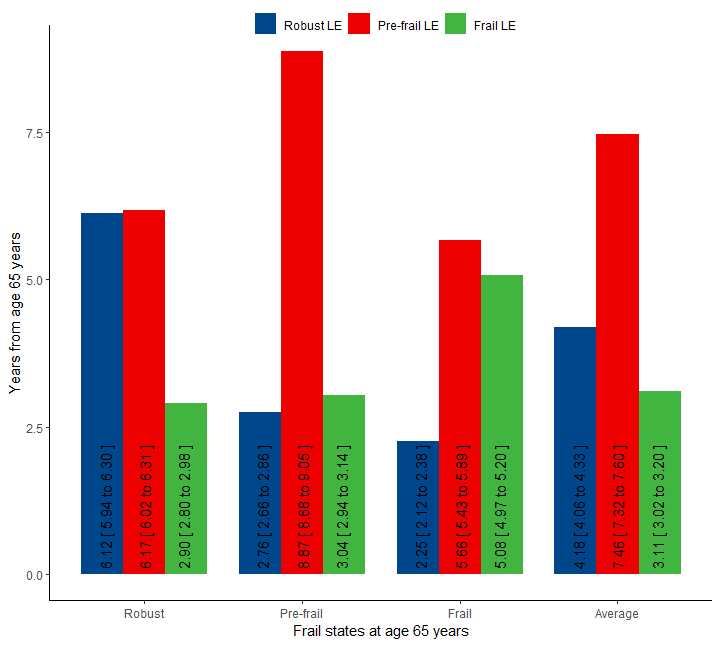


**Fig. S21. Life expectancies according to starting state at age 65 years**

**(Results of** **frailty index excluding cancers)**


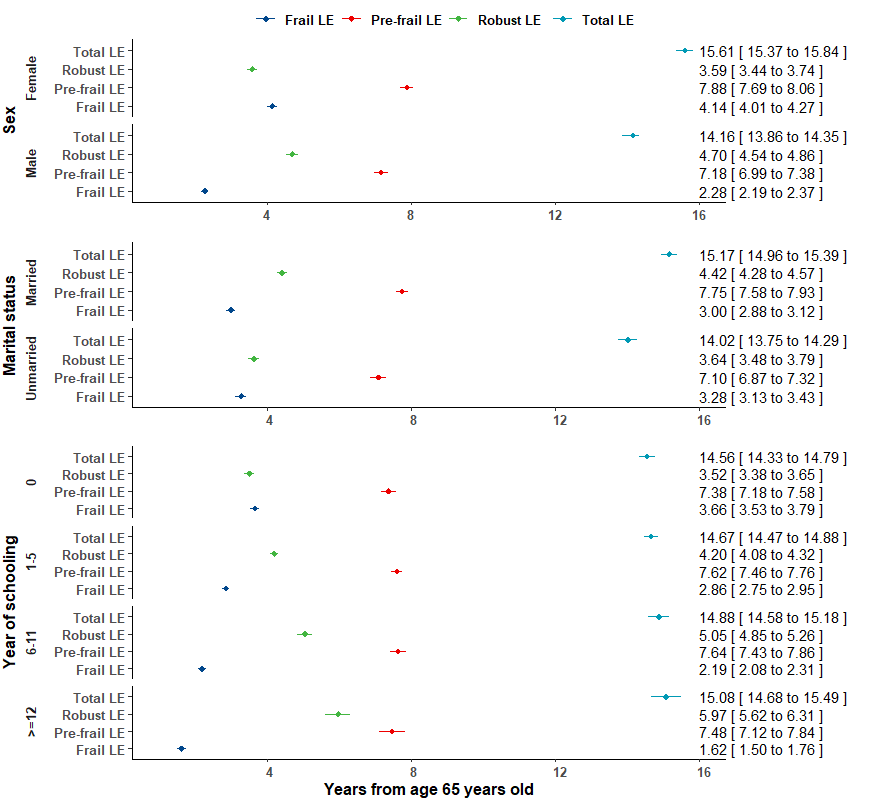


**Fig. S22. Life expectancies by sex, marital status and years of schooling**

**(Results of** **frailty index excluding cancers)**


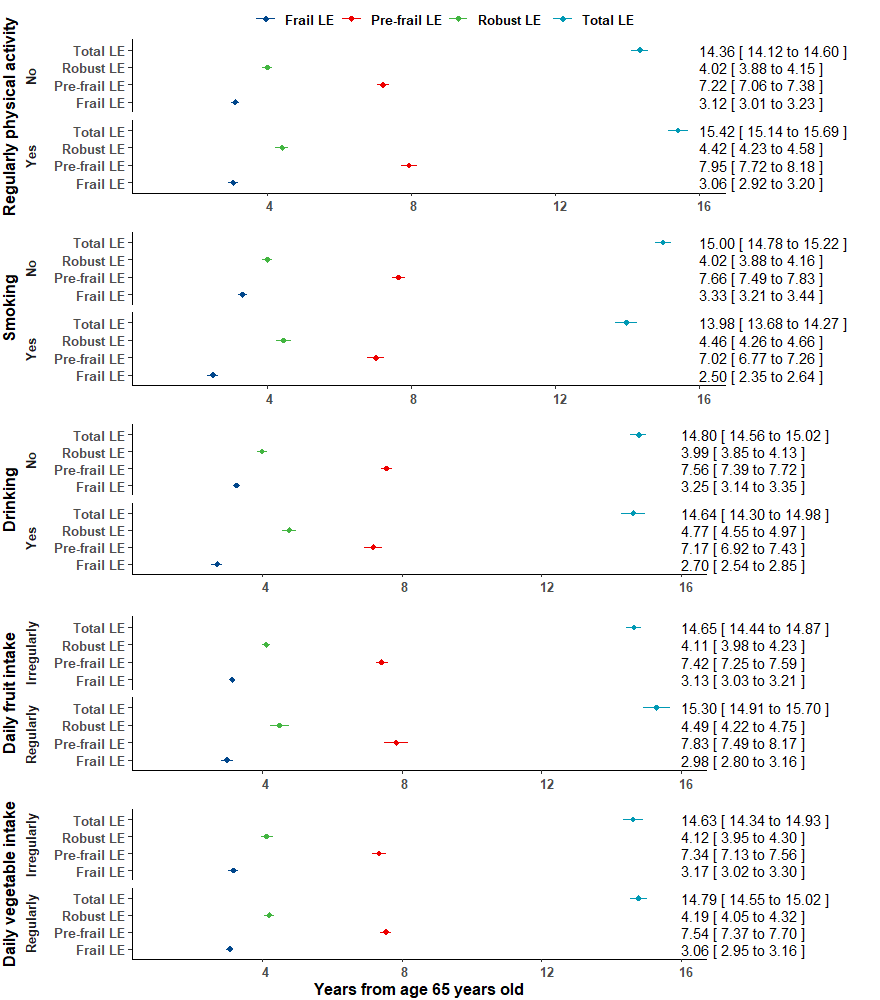


**Fig. S23. Life expectancies by behaviours**

**(Results of** **frailty index excluding cancers)**


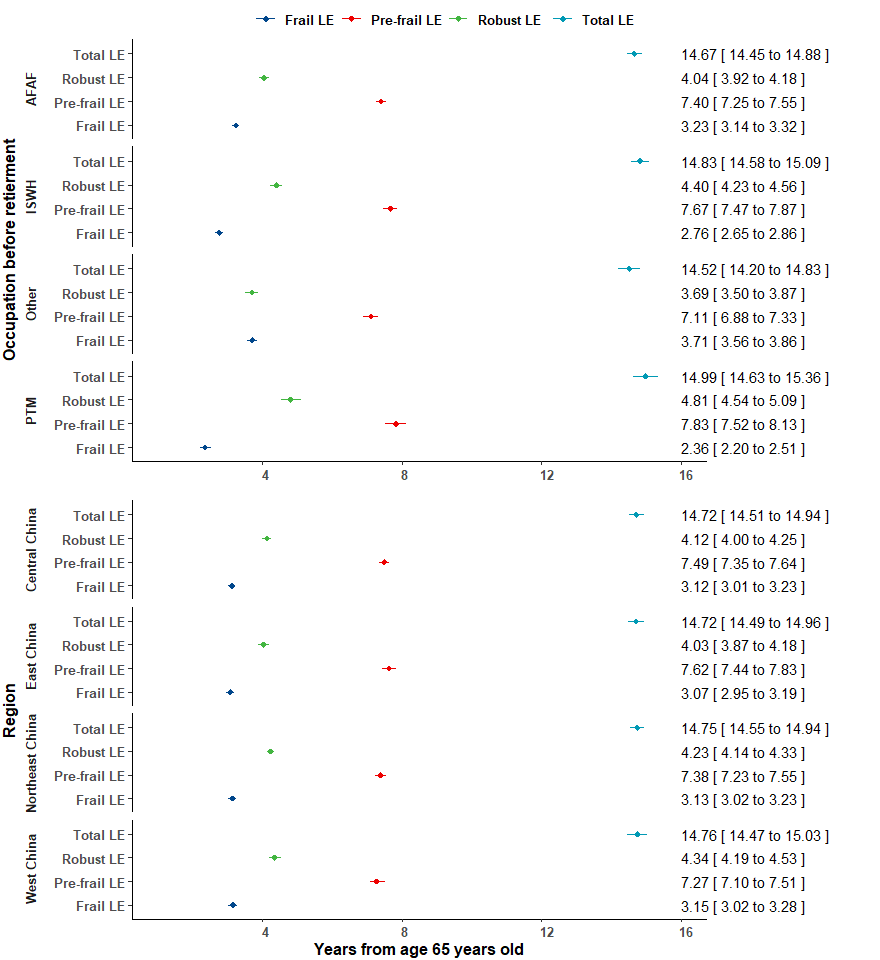


**Fig. S24. Life expectancies by occupations and regions**

**(Results of** **frailty index excluding cancers)**


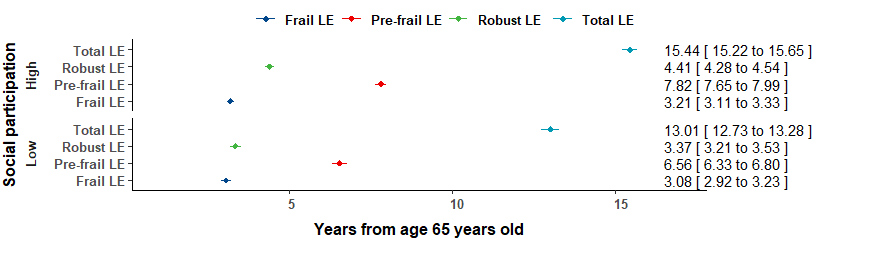
 **Fig. S25. Life expectancies by social participation**

**(Results of** **frailty index excluding cancers)**
